# Forty years later: adult health and non-communicable disease following the 1984–1985 Great Ethiopian Famine – a retrospective cohort study

**DOI:** 10.1136/bmjgh-2025-021721

**Published:** 2026-02-23

**Authors:** Mubarek Abera, Gemechu Ameya, Melkamu Berhane, Carlos Salvador Grijalva-Eternod, Natasha Lelijveld, Grace O Donovan, Elizabeth Wimborne, Kenneth Anujuo, Amir Kirolos, Olga Laurentya, Kimberley McKenzie, Benedikte Grenov, Mekitie Wondafrash, Gerard Bryan Gonzales, Tsinuel Girma, Alemseged Abdissa, Debbie Thompson, Charles Opondo, Tim Cole, Albert Koulman, Jonathan Swann, Jonathan CK Wells, Marko Kerac, CHANGE Study Collaborators Group, Minale Fekade

**Affiliations:** 1 Department of Psychiatry, Institute of Health, Jimma University, Jimma, Ethiopia; 2 Jimma University Clinical and Nutrition Research Center (JUCAN), Jimma University, Jimma, Ethiopia; 3 Department of Laboratory Science, Menelik II Medical and Health Science College, Addis Ababa, Ethiopia; 4 Department of Pediatrics and Child Health, Institute of Health, Jimma University, Jimma, Ethiopia; 5 Population Health, London School of Hygiene & Tropical Medicine, London, UK; 6 Institute for Global Health, University College London, London, UK; 7 Emergency Nutrition Network, Kidlington, UK; 8 School of Human Development and Health, University of Southampton, Southampton, UK; 9 Medical Research Council, Epidemiology Unit, University of Cambridge, Cambridge, UK; 10 Department of Women and Children’s Health, Institute of Life Course and Medical Sciences, University of Liverpool, Liverpool, UK; 11 Core Metabolomics and Lipidomics Laboratory, University of Cambridge, Cambridge, UK; 12 Caribbean Institute for Health Research, The University of the West Indies, Kingston, Jamaica; 13 Department of Nutrition, Exercise and Sports, Københavns Universitet, Kobenhavn, Denmark; 14 St. Paul Institute for Reproductive Health and Rights, Addis Ababa, Ethiopia; 15 Public Health and Primary Care, Ghent University, Gent, Belgium; 16 Jimma University, Jimma, Ethiopia; 17 Armaur Hansen Research Institute, Addis Ababa, Ethiopia; 18 Department of Medical Statistics, London School of Hygiene and Tropical Medicine Faculty of Epidemiology and Population Health, London, UK; 19 Nuffield Department of Population Health, University of Oxford, Oxford, UK; 20 Population, Policy and Practice Programme, UCL, London, UK; 21 MRC Epidemiology Unit, University of Cambridge, Cambridge, UK; 22 Childhood Nutrition Research Centre, Population, Policy and Practice Department, UCL Great Ormond Street Institute of Child Health, London, UK

**Keywords:** Africa, Aging, Global Health, Stunting, Health policies and all other topics

## Abstract

**Background:**

As the threat of child malnutrition increases, the focus remains mostly on short-term consequences. Long-term sequelae are increasingly recognised but lack strong evidence, and many studies face methodological limitations.

**Method:**

A retrospective cohort of survivors of the 1984–1985 Great-Ethiopian Famine was compared with two novel control groups: born post-famine; and age category- and sex-matched controls. Exposure to famine at different age categories was explored (fetal, 0–2, 2–5, 5–10 and 10–18 years). Follow-up was 40 years later. Outcomes included anthropometry, body composition, arterial stiffness, mental health, and risk of cardiometabolic and non-communicable diseases (NCDs). Adjusted differences and 95% CI between exposed and controls were calculated.

**Results:**

Compared with matched and post-famine controls, adjusted differences (95% CI) for exposed group were: height, −1.4 cm (−2.4 to –0.3) and −2.4 cm (−3.7 to –1.1); weight, −1.4 kg (−2.7 to –0.1) and −1.7 kg (−3.3 to –0.1); diastolic blood pressure (DBP), −2.8 mm Hg (−4.4 to –1.1) and 2.8 mmHg (0.9 to 4.7); handgrip strength, −1.7 kg (−2.7 to –0.6) and −4.1 kg (−5.5 to –2.7); brachial augmentation index, 5.4% (0.3% to 10.5%) and 16.1% (10.1% to 22.1%); aortic augmentation index, 6.0% (1.5% to 10.4%) and 11.7% (6.1% to 17.3%); subscapular skinfold thickness, 1.1 mm (0.2 to 1.9) and 1.2 mm (0.1 to 2.3); triceps skinfold thickness, 1.8 mm (0.8 to 2.7) and 2.1 mm (1.0 to 3.3) and waist-to-height ratio, 0.01 (0.003 to 0.02) and 0.01 (0.001 to 0.02), respectively. When comparing risk by timing of exposure, individuals exposed during early childhood (0–2 years), preschool age (2–5 years), and late childhood (5–10 years) had reduced adult stature of −2.8 cm (–4.8 to –0.9), −2.8 cm (−4.7 to -0.9) and −2.1 cm (–4.0 to −0.2), respectively, and increased triceps skinfold of 1.7 mm (−0.5 to 3.8), 3.2 mm (0.8 to 5.6) and 3.8 mm (1.6 to 6.02), respectively.

**Conclusions:**

Early-life famine exposure is associated with smaller adult size and several, but not all NCD risks. Lower DBP in survivors compared with matched controls is surprising and might reflect differential susceptibility to specific later-life health risks. Greater arterial stiffness underscores the need to identify both preclinical and clinical risk. In contrast to exposure in utero, risk was higher among those exposed during early childhood (0-2 years), preschool (2–5 years) and late childhood (5–10 years). The study underscores the need for a dual approach in low- and middle-income settings: tackling the immediate undernutrition while also anticipating and mitigating long-term NCD risk in populations exposed to early-life severe malnutrition or famine.

**WHAT IS ALREADY KNOWN ON THIS TOPIC**

- Our recent systematic review shows early-life famine or malnutrition is associated with adult non-communicable disease (NCD) risk. However, many studies have key limitations. Notably, most focused on famine exposure in the first 1000 days, used controls born post-famine (which induces age-related bias), many did not consider the exact age at exposure (hence exposure classification is oversimplified), and few studies assessed early subclinical markers of NCD risk.

**WHAT THIS STUDY ADDS**

- We established a migrant-based cohort of famine survivors with two distinct novel control groups (age and sex-matched, and post-famine) and assessed clinical NCD risk and arterial stiffness as future hypertension and cardiovascular disease markers. For the first time, we show higher arterial stiffness in famine survivors compared with both control groups, whereas diastolic blood pressure in survivors was higher than in post-famine controls, but lower than in matched controls.

**HOW THIS STUDY MIGHT AFFECT RESEARCH, PRACTICE OR POLICY**

- Our results underscore the need for robust control groups and inclusion of subclinical markers to better understand famine-associated NCD risk. Post-famine NCD is influenced by early life phenotypic predisposition and later-life exposures, requiring longer-term follow-up to truly understand impact. Innovative health systems should be considered in drought and famine-affected regions.

## Introduction

 Global food insecurity is on the rise due to climate change, conflict and migration^1^; this will worsen as international funding and aid becomes increasingly strained. The 1980s Great Ethiopian Famine was one of the worst famines in the 20th century.^2^ It severely affected 20% of the country’s population, leaving 300 000 to 1.2 million dead, and 2.5 million internally displaced.^3^ As the global threats to child malnutrition continue to rise, due to factors such as climate change, economic shocks, inequity, conflict, instability, forced displacement and decreasing international aid,^4 5^ most attention remains focused on the short-term consequences, particularly the risk of death. Long-term sequelae are increasingly recognised, yet evidence is sparse and many existing studies face methodological limitations.

**Figure 1:**
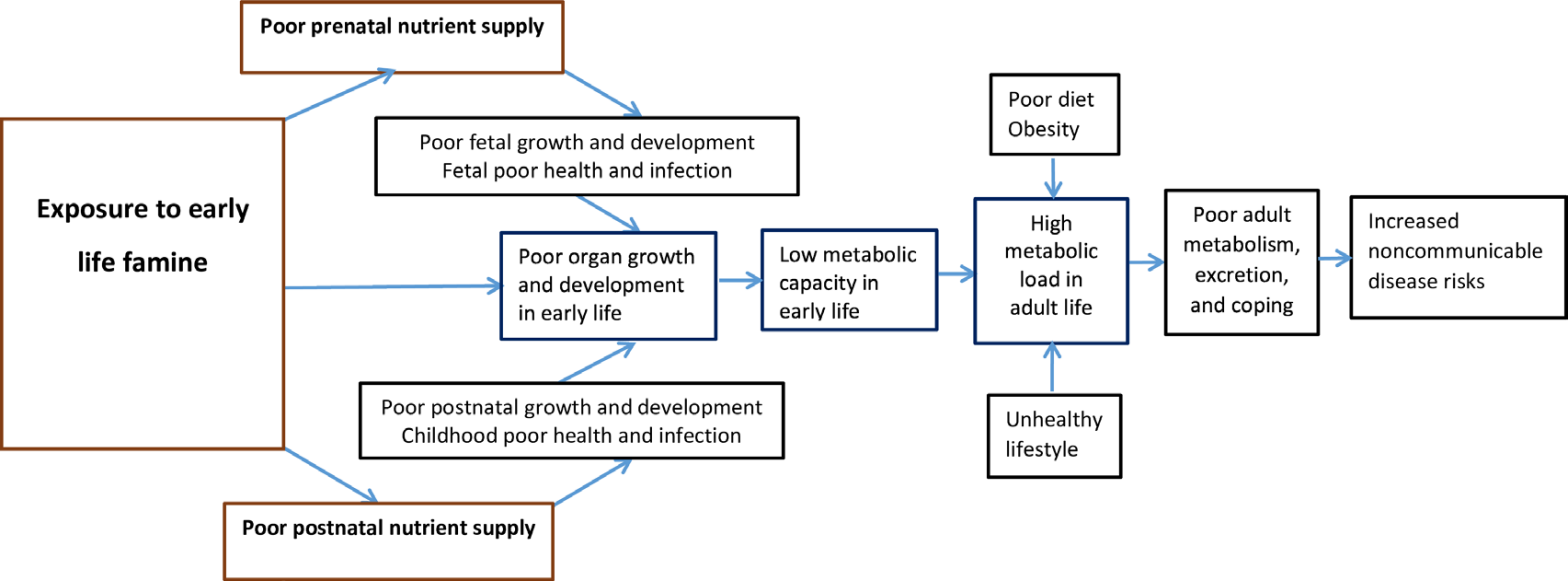
Conceptual framework linking early life malnutrition with later health and disease outcomes in the Ethiopian 1980s famine cohort based on the thrifty phenotype and capacity load model.

In the 1980s, Barker reported associations between low birth weight and adult coronary heart disease for the first time,^6 7^ leading to the developmental origins of adult health and disease hypothesis.^8^ In the 1990s and early 2000s, the long-term consequences of childhood stunting have been documented.^9^ Since then, a growing body of research has also shown the long-term consequences of severe malnutrition or famine exposure during early life, such as poor physical, mental and cardiometabolic health. This has been documented in different parts of the world^10^,^12^; notably in the Dutch Hunger Winter^13^,^15^ and the Chinese famine.^16^,^20^ Exact mechanisms linking early-life exposure to famine/malnutrition and later-life risks for non-communicable disease (NCD), however, remain elusive. Current explanations of how early-life malnutrition impacts long-term health include the thrifty phenotype hypothesis^21^ and the capacity-load model.^22^ Moreover, literature on the long-term consequences of famine or childhood malnutrition has several limitations, paying inadequate attention to the age of individuals at the time of exposure to the famine or malnutrition and the age of the control groups.^11^ Thus, in this study, we aimed to examine the effects of exposure to famine during different early-life periods (specifically in utero; 1st 1000 days; 2–5 years (preschool); 6–10 years (school-age); 10–18 years (adolescent) on later-life health outcomes, while also exploring how the risks vary, depending on the choice of control groups. Our conceptual framework is presented in figure 1.

Our hypotheses are that:

1. Individuals exposed to famine during early life (from fetal to adolescence) have an increased risk of adverse health outcomes and NCD risk in adulthood.
2. Age of individuals at the time of exposure (fetal, 0–2 years, 2–5 years, 5–10 years and 10–18 years) shapes the magnitude of the effect on health and NCD outcomes in adult life.
3. The choice of unexposed control group is important, with risks differing depending on whether the comparison is with controls born post-famine or age- and sex-matched controls.

The findings of this study will provide robust evidence on how early-life malnutrition or famine exposure affects later-life physical, mental and cardiometabolic health outcomes, offering critical insights to guide nutrition interventions, health system planning and policies aimed at reducing the long-term consequences of severe malnutrition.

### Methods

### Setting and period

This community-based study was conducted from 5 January to 30 March 2024, in Jimma, Southwest Ethiopia. In consultation with national and regional authorities, we identified the areas where survivors of the 1980s famine had been relocated. We then engaged with the Jimma Zone authorities to determine the specific administrative units in which these relocated survivors currently reside. Jimma is famine-unaffected area to which survivors of the 1984–1985 famine (which mainly affected Northern areas of the country) were relocated and settled during the immediate post-famine period. Residents in this area are primarily farmers who cultivate grain (maize, teff and rice), coffee, as well as roots and fruits. Though individual level household food insecurity may exist, the study setting at community level is a food-secure and productive area. Consequently, both the exposed (after relocation) and control groups have had adequate food access and did not require any food aid or Safety Net support.

### Design

We established a historical cohort of individuals exposed to famine using a novel design, with two complementary control groups. The first control group was a native Jimma population matched by sex and age category at the time the famine occurred: in utero, early childhood (0–2 years), preschool (2–5 years), late childhood (5–10 years) and adolescence (10–18 years) (referred to as matched controls). We also included a second control group born post-famine to parents who were relocated from the famine-affected area to Jimma during or immediately after the famine, and born to famine non exposed parents in the local community - (referred to as post-famine controls). The data was collected through home-to-home visits using trained data collectors.

### Population

The population consisted of famine-exposed and non-exposed individuals recruited through home-to-home visits in the community. In collaboration with the zonal and district administrations, we identified local residents—matched controls—who were born and raised in the Jimma Zone. Daily community-based data collection was supported by local community guides. To establish exposure status, before enrolment, all participants were screened using questions on their place and date of birth, the area where they grew up, exposure to the 1980s northern famine and exposure to any other famine events. Pregnant women and individuals with acute severe illness who were unable to communicate or required urgent medical attention were excluded. The date of birth is provided in online supplemental figure 1.

### Exposure and exposed group

The exposure of interest is the 1984–1985 Great Ethiopian Famine that occurred in the northern part of the country. The famine was characterised by a severe shortage or complete lack of food following an extended drought impacting harvesting that resulted in widespread starvation, severe malnutrition and high mortality. The geographic coverage of the famine has been documented by both the national government and international organisations, indicating that the most affected areas were Tigray, Wollo and the northern part of Shewa. Our study populations are those individuals who survived the 1984–1985 famine and their controls. The famine survivors had been exposed between September 1984 and August 1985 and were divided into subgroups based on their age at the time of exposure. To be classified as exposed, individuals had to meet all three of the following criteria: (A) resided in a drought-affected or famine-affected area during 1984–1985; (B) was in utero, or between the ages of 0 and 18 years, during the famine (ie, born between September 1966 and May 1986) and (C) experienced famine conditions for at least 1 month.

### Control groups

All controls were individuals who had not been exposed to the 1980s Great Ethiopian Famine or any other famine in their lifetime. Controls were selected from the Jimma area based on screening procedures designed to minimise the likelihood of prior exposure to famine or severe childhood malnutrition. For matched controls, the following three criteria had to be met: (A) never lived in the drought/famine-affected areas; (B) not exposed to the 1984–1985 drought/famine or any other famine and (C) born between September 1966 and May 1986. The other group of controls, born post-famine, had to be born in the Jimma area between September 1987 and December 1993. Controls born post-famine had two subgroups. The first subgroup consisted of those born post-famine to exposed parents who had migrated from the famine area to Jimma, and the second subgroup were those born to non-exposed parents in the local Jimma community. These screening steps were used to ensure that control participants were unlikely to have experienced major nutritional shocks during childhood.

### Outcomes of interest

Several outcome indicators were collected to assess physical health, mental health and NCD risk. These included adult anthropometry (height, weight, body mass index (BMI), mid-upper arm circumference), handgrip strength, body composition (fat mass, visceral tissue and skeletal muscle mass), measures of central adiposity (waist circumference, hip circumference, waist-to-hip ratio, and waist-to-height ratio), skinfold thicknesses (triceps and subscapular), blood pressure and arterial stiffness. We also assessed episodes of non-communicable chronic illness and mental illness outcomes.

### Covariates

To account for potential confounding factors, we collected data on age, sex, educational status, occupation, religion and wealth status, which were included in statistical analysis.

### Measurements and tools

A structured questionnaire was developed to collect data on sociodemographic and economic conditions, medical history and pregnancy-related history (females only). Sociodemographic and economic information included age, sex, education, occupation, marital status, religion and relative economic position compared to the local community. Data on medical history covered previous diagnosis of hypertension, diabetes, epilepsy, cardiovascular diseases, stroke, asthma, chronic pain, mental illness and suicide risk. These were assessed using closed-ended (yes/no) questions. The current status of hypertension, anxiety and depression was assessed using blood pressure measurement and reported symptoms.

Current mental health status was assessed using the Patient Health Questionnaire-9 for depression^23^ and the Generalised Anxiety Disorder-7 questionnaire for anxiety problems.^24^ Lifestyle- and behaviour-related factors were also assessed, including alcohol and substance use (Khat, Nicotine, Marijuana, Cannabis or Cocaine) for life-time, past 12 months and current use; physical activity (self-reported using the Global Physical Activity Questionnaire for a typical week)^25^; dietary patterns (self-reported 24-hour recall using the Global Diet Quality Questionnaire)^26^ and sleep quality, assessed using a standardised questionnaire.^27^

### Anthropometry/physical measurements

Anthropometric indicators were measured following the WHO growth assessment protocol,^28 29^ employing two independent measurers to obtain paired anthropometric measurements. If the two measurements differed beyond the acceptable range, both observers carried out a repeated measurement. Height was measured using an adult height board to the nearest 0.1 cm, and weight using a digital Seca 286 weighing scale to the nearest 0.1 kg. Waist and hip circumferences were measured to the nearest 0.1 cm using a SECA girth tape (Seca, Germany, Model 200). Handgrip strength in kg was measured using a Dynamometer (Jamar). Systolic and diastolic blood pressure (SBP and DBP) were measured to the nearest mm Hg after a 5 min rest using an Omron BP785 digital blood pressure device. Hypertension was defined by SBP of 140 mm Hg or higher, or DBP of 90 mm Hg or higher. Arterial wall stiffness was measured using pulse wave velocity analysis (Arteriograph, TensioMed).

Body composition (total fat mass, visceral fat mass and skeletal muscle mass) was predicted using a portable non-invasive bio-electrical impedance device using manufacturers’ equations (Omron Body Composition Scanner). Skinfold thickness was measured using a Harpenden calliper (Baty International, England) at the subscapular and triceps areas in duplicate.

Quality assurance: The research team completed 1 week of training and a pilot test, with standardised procedures to ensure consistency. Additional quality was maintained through spot checks, double measurements, double data entry and continuous supervision by field coordinator and supervisors.

### Sample size considerations

Assuming a mean difference of 2.5 mm Hg with 5.0 mm Hg standard deviation (SD) DBP from a previous study^30^ and a 2:1 ratio between exposed and control groups, with 80% power, 95% confidence level, the required sample size was 94 in each exposed age-category group and 47 for each control group. Inflating by 15% to account for potential non-response or missing data, a total of 805 participants were recruited, with 107 participants in each exposed age-category group, and 54 participants in each control group, across all five age categories. Likewise, the post-famine control groups consisted of 107 individuals born post-famine to migrant famine-exposed parents; and 54 born to famine non-exposed local parents, totalling 161 participants. We employed a consecutive sampling method. In all administrative units where participants were enrolled, screening and recruitment started from the centre of the administrative unit and moved out to the periphery until we had reached our target sample size. Only one participant was recruited from each selected household.

### Statistical analysis

Electronically collected data were exported to STATA release V.18.5 (StataCorp). All anthropometric measurements were calculated as the arithmetic mean of duplicate measurements. Categorical data were analysed and described using percentage and frequency while continuous data were analysed using either mean and SD or median and IQR after checking the normality of the distribution. Lifetime NCD episodes were defined by the reported presence of any of the following: hypertension, diabetes, raised cholesterol, stroke, mental health problems, epilepsy and any other chronic NCD.

Analyses were conducted using standard linear regression for continuous outcomes, and logistic regression for binary outcomes, with prespecified models. As the matching was done by frequency for age category rather than by paired (one-to-one) age-matching, we employed a standard regression analysis model where the analysis was adjusted for age, rather than being adjusted for matching as with conditional regression analysis model. Models were developed based on confounding factors described in the literature and the presence of biologically plausible relationships. Model 1 was unadjusted, model 2 adjusted for age and sex and model 3 further adjusted for religion, educational status and socioeconomic status. Additionally, Model 3 included current BMI, alcohol and substance use, physical activity and diet quality when outcomes were blood pressure, skinfolds, body composition, hip and waist circumferences, handgrip strength and mental health problems. However, current BMI was excluded and height was added into model 3 when outcomes were body fat, visceral fat and skeletal muscle mass. Finally, the adjusted difference (AD) in relative risk or coefficient estimates and 95% CIs was reported.

## Results

### Background characteristics

Of 986 individuals screened, 966 consented to participate. Among these, 528 (55%) were in the exposed group, 280 (29%) were matched controls and 158 (16%) post-famine controls. The study flow chart is presented in online supplemental figure 2. The age range was 38–57 years for the exposed group and their matched controls, and 31–36 years for the post-famine controls (table 1).

**Table 1:** Sociodemographic and anthropometry characteristics of the 1980s Great Ethiopian Famine cohort by exposure status after 40 years

Anthropometric and clinical characteristics		Exposed	Matched control	Born post-famine		
		N=528	N=280	All born post-famine n=158	Born to famine exposed parent; n=104	Born to famine non-exposed parent; n=54
Age in years, mean (SD)		43.9 (5.7)	43.8 (5.7)	32.1 (2.1)	32.2 (2.2)	31.9 (2.1)
Sex, female, n (%)		252 (48.4)	133 (47.7)	85 (54.1)	55 (51.9)	30 (58.8)
Religion, n (%)	Orthodox Christian	321 (60.8)	31 (11.1)	89 (56.7)	83 (79.8)	6 (11.1)
	Muslim	203 (38.4)	249 (88.9)	64 (40.8)	21 (20.2)	44 (81.5)
	Other	4 (0.8)	0 (0)	4 (2.6)	0 (0)	4 (7.4)
Ever attended school	Yes (n (%))	217 (41.1)	140 (50.0)	94 (57.0)	57 (54.8)	37 (68.5)
Educational status among those ever attended school, n (%)	Illiterate	17 (7.8)	9 (6.4)	4 (4.3)	3 (5.3)	1 (2.7)
	Able to read/write	17 (7.8)	7 (5.0)	2 (2.1)	1 (1.8)	1 (2.7)
	Grade 1–6	159 (73.3)	99 (70.1)	44 (46.8)	28 (49.1)	16 (43.2)
	Grade 7–8	21 (9.7)	13 (9.3)	9 (9.6)	7 (12.3)	2 (5.4)
	Grade 9–12	3 (1.4)	10 (7.1)	21 (22.3)	14 (24.6)	7 (18.9)
	Diploma	0 (0)	2 (1.4)	5 (5.3)	1 (1.8)	8 (21.6)
	Degree	0 (0)	0 (0)	0 (0)	3 (5.3)	2 (5.4)
Anthropometry measurements, mean (SD)	Height (cm)	160.2 (8.3)	161.8 (8.3)	162.3 (9.6)	163.2 (9.4)	160.4 (9.8)
	Sitting height (cm)	81.3 (5.4)	81.1 (4.9)	81.6 (4.7)	82.2 (4.3)	80.1 (5.0)
	Weight (kg)	51.3 (8.2)	52.1 (8.4)	52.8 (8.8)	53.3 (8.9)	51.8 (8.5)
	BMI (kg/m^2^)	20.0 (2.8)	19.9 (2.8)	20.0 (2.6)	20.0 (2.6)	20.1 (2.6)
	MUAC (cm)	24.5 (2.8)	24.1 (2.6)	24.4 (2.3)	24.4 (2.2)	24.3 (2.5)
Categorical BMI, n (%)	Thin	66 (12.5)	31 (11.1)	15 (9.5)	12 (11.5)	3 (5.6)
	Underweight	99 (18.8)	60 (21.4)	21 (13.3)	10 (9.6)	11 (20.4)
	Normal	331 (62.7)	176 (62.9)	113 (71.5)	77 (74.0)	36 (66.7)
	Overweight	24 (4.5)	11 (3.9)	9 (5.7)	5 (4.8)	4 (7.4)
	Obese	8 (1.5)	2 (0.7)	0 (0)	0 (0)	0 (0)
Markers of body composition and adiposity, mean (SD)	Body fat mass %	21.1 (9.9)	20.7 (10.4)	22.2 (9.4)	21.4 (8.9)	23.7 (10.3)
	Body fat, in kg	10.8 (1.7)	10.8 (1.7)	11.7 (2.0)	11.4 (1.8)	12.3 (2.1)
	Visceral fat %	4.1 (2.9)	3.8 (2.2)	3.4 (1.6)	3.4 (1.6)	3.3 (1.5)
	Visceral fat, kg	2.1 (0,3)	2.0 (0.3)	1.8 (0.3)	1.8 (0.3)	1.7 (0.3)
	Skeletal mass %	33.2 (7.1)	33.6 (7.0)	33.6 (7.3)	34.3 (6.9)	32.4 (7.9)
	Skeletal mass, kg	17.1 (2.7)	17.5 (2.8)	17.7 (3.0)	18.3	16.8 (2.6)
	Waist circumference (cm)	75.4 (7.9)	74.5 (7.9)	74.3 (7.4)	74.2 (7.4)	74.4 (7.5)
	Hip circumference (cm)	87.2 (6.8)	87.0 (6.3)	87.8 (6.5)	88.1 (6.6)	87.0 (6.4)
	Waist-to-hip ratio	0.87 (0.1)	0.87 (0.07)	0.85 (0.06)	0.85 (0.06)	0.85 (0.07)
	Waist-to-height ratio	0.47 (0.05)	0.46 (0.05)	0.46 (0.05)	0.46 (0.05)	0.46 (0.05)
	Subscapular skinfold (mm)	11.2 (6.1)	9.9 (4.8)	10.0 (5.6)	9.6 (3.6)	
	Triceps skinfold (mm)	11.0 (6.9)	9.2 (5.6)	9.3 (5.3)	8.8 (5.1)	10.8 (7.7)
Handgrip strength, mean (SD)	Measured in (kg)	20.9 (7.7)	23.2 (7.5)	25.0 (8.4)	26.0 (8.6)	23.1 (7.8)
Blood pressure, mean (SD)	SBP (mm Hg)	113.6 (13.4)	116.9 (18.3)	111.7 (13.1)	112.7 (12.0)	109.6 (15.0)
	DBP (mm Hg)	77.6 (9.2)	80.1 (10.9)	74.2 (9.6)	74.0 (9.0)	74.5 (10.8)
	Hypertension, n (%)	69 (12.7)	50 (17.7)	9 (5.7)	5 (4.8)	4 (7.4)
Arterial stiffness, mean (SD)	Aix brachial—Brachial Augmentation Index (%)	−12.5 (32.8)	−16.9 (28.2)	−34.5 (23.7)	−37.5 (22.6)	−28.6 (24.9)
	Aix aortic—Aortic Augmentation Index (%)	32.2 (31.0)	29.1 (14.6)	19.6 (11.2)	18.1 (10.7)	22.3 (11.5)
	Ejection duration of the left ventricle (ms)	289.6 (39.7)	289.9 (32.1)	291.5 (26.7)	290.8 (29.5)	292.8 (20.4)
	Pulse wave return time (ms)	123.1 (25.3)	118.6 (24.9)	136.4 (24.0)	138.2 (24.6)	133.1 (22.6)
	Aortic pulse wave velocity (m/s)	10.9 (16.3)	11.3 (15.2)	8.2 (5.3)	8.4 (6.4)	7.9 (1.4)
	PPao—Central pulse pressure (mm Hg)	48,4 (15.4)	47.7 (22.8)	45.6 (33.4)	47.4 (40.6)	42.1 (9.3)
	SBPao—Central SBP (mm Hg)	120.5 (59.7)	124.7 (66.0)	120.0 (115.8)	123.8 (142.4)	112.7 (14.5)
	Sys—Brachial systolic blood pressure (mm Hg)	121.6 (15.6)	124.5 (18.4)	120.1 (13.9)	120.5 (13.4)	119.2 (14.9)
	Dia—Brachial diastolic blood pressure (mm Hg)	72.1 (10.7)	76.1 (14.0)	70.0 (9.8)	69.5 (9.1)	71.0 (11.1)
	Mean arterial pressure (mm Hg)	88.5 (11.3)	92.1 (14.2)	85.9 (11.4)	86.5 (9.5)	84.7 (14.3)
	Brachial pulse pressure (mm Hg)	49.9 (10.6)	49.1 (11.0)	50.6 (10.8)	51.3 (10.8)	49.3 (10.8)
	Heart rate	72.8 (11.2)	73.4 (10.9)	73.6 (12.3)	72.9 (13.1)	74.9 (10.4)
NCD markers	Mild to severe depression, n (%)	47 (8.9)	24 (8.6)	6 (4.4)	7 (6.7)	0 (0)
	Mild to severe anxiety, n (%)	23 (4.6)	9 (3.1)	3 (1.9)	3 (2.9)	0(0)
	Life time episodes of NCD, n (%)	47 (8.9)	28 (10.0)	5 (3.2)	2 (1.9)	3 (5.6)

BMI, body mass index; DBP, diastolic blood pressure; MUAC, mid-upper arm circumference; NCD, non-communicable disease; SBP, systolic blood pressure.

### Anthropometric, body composition and clinical profiles

Mean and SD for height and weight were 160.2 (8.3) cm and 51.3 (8.2) kg in the exposed, and 161.8 (8.7) cm and 52.1 (8.4) kg in the matched controls. Mean blood pressure was 113.6 (13.4) mm Hg and 77.6 (9.2) mm Hg for SBP and DBP in the exposed, and 116.9 (18.3) mm Hg and 80.1 (10.9) mm Hg in the matched controls. Handgrip strength was 20.9 (7.7) kg in the exposed and 23.2 (7.5) kg in the matched controls. In the unmatched (postfamine) controls, individuals born to famine-exposed parents had an average height of 163.2 cm, weight of 53.3 kg, SBP of 112.7 mm Hg, DBP of 74.0 mm Hg, handgrip strength of 26.0 kg, body fat percentage of 21.4% and skeletal mass percentage of 34.3%. Those born to non-exposed parents had an average height of 160.4 cm, weight of 51.8 kg, SBP of 109.6 mm Hg, DBP of 74.5 mm Hg, handgrip strength of 23.1 kg, body fat percentage of 23.7%, and skeletal mass percentage of 32.4%. Additional age-stratified clinical data are presented in table 1.

The prevalence of depression and anxiety was 47 (8.9%) and 23 (4.6%), respectively, in the exposed group, and 24 (8.6%) and 9 (3.1%) in the matched controls. Lifetime episodes of any forms of NCD (other than mental illness) were 47 (8.9%) and 28 (10%) in the exposed and matched control groups, respectively.

### Risk of poor health and NCD in famine-exposed groups by age at the time of exposure

**Figure 2:**
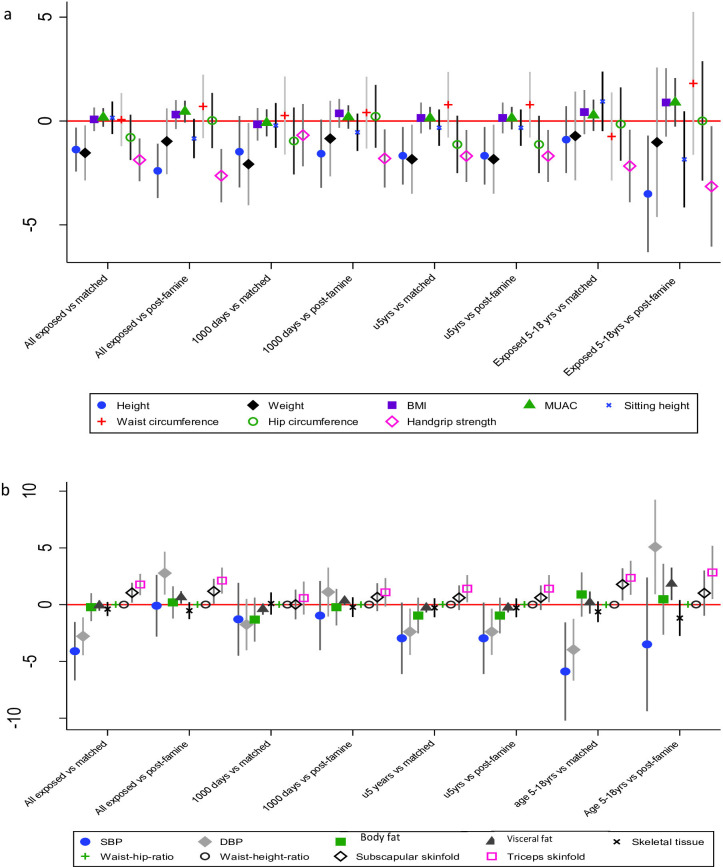
Effects of famine exposure on anthropometry (**a**) and NCD risk (**b**) in all exposed, in the first 1000 days and in u5 years exposed groups. BMI, body mass index; DBP, diastolic blood pressure; MUAC, mid-upper arm circumference; NCD, non-communicable disease; SBP, systolic blood pressure; u5, under 5.

In adjusted regression models, the overall AD and 95% CI for the exposed group in height was −1.4 cm (−2.4 to –0.3) and −2.4 cm (−3.7 to −1.1) compared with matched and post-famine controls, respectively. The AD in weight was −1.4 kg (−2.7 to –0.1) and −1.0 kg (−2.6 to 0.6) for the exposed compared with matched and born post-famine controls. In comparison to matched and post-famine controls, the AD for the exposed group in subscapular skinfold was 1.1 mm (0.2 to 1.9) and 1.2 mm (0.1 to 2.3), triceps skinfold was 1.8 mm (0.8 to 2.7) and 2.1 mm (1.0 to 3.3), handgrip strength was −1.7 kg (−2.7 to –0.6) and −4.1 kg (−5.5 to –2.7), and waist-to-height ratio was 0.01 (0.003 to 0.02) and 0.01 (0.001 to 0.02), respectively. Compared with matched and born-post famine controls, the AD in the exposed group for SBP was 4.1 mm Hg (−6.7 to –1.5) and −0.1 mm Hg (−2.8 to 2.6), and DBP was −2.8 mm Hg (−4.4 to –1.1) and 2.8 mm Hg (0.9, 4.7), respectively. Details are provided in figure 2 and online supplemental table 1.

Compared with matched and post-famine controls, AD of the exposed group during the first 1000 days in height was −1.5 cm (−3.2 to 0.2) and −1.6 cm (−3.2 to 0.1) and weight was −2.1 kg (−4.1 to –0.1) and −0.9 kg (−2.7 to 1.0), respectively. AD in waist-to-height ratio was 0.01 (0.0 to 0.02) and 0.01 (−0.003 to 0.02) and handgrip strength was −0.7 kg (−2.2 to 0.8) and −1.8 kg (−3.2 to –0.4) in the exposed compared with matched and post-famine controls (see online supplemental table 2 for detail).

**Figure 3:**
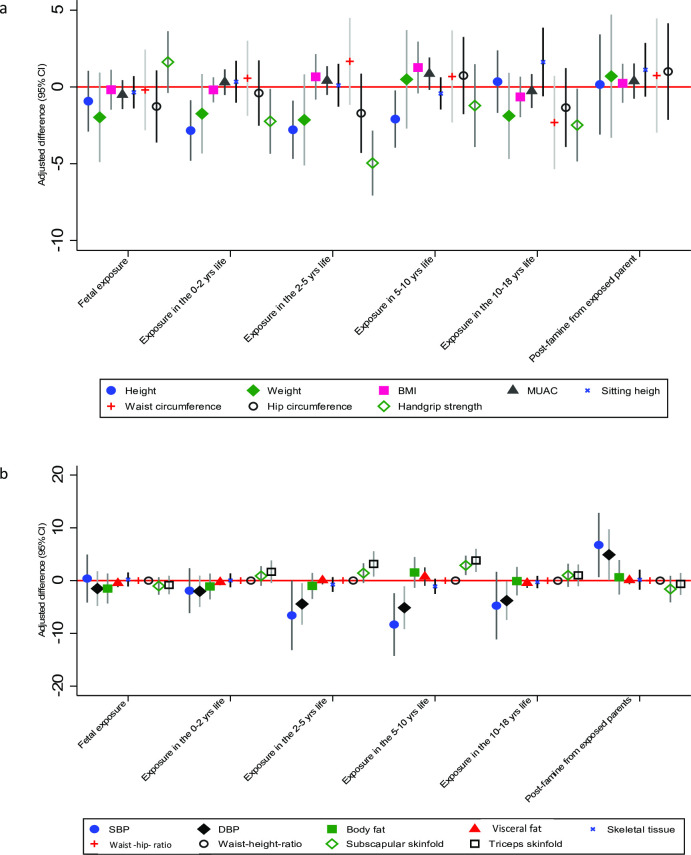
Effects of famine exposure on anthropometry (**a**) and NCD risk (**b**) in six different chronological age exposure groups compared with their matched controls. BMI, body mass index; DBP, diastolic blood pressure; MUAC, mid-upper arm circumference; NCD, non-communicable disease; SBP, systolic blood pressure.

AD of the exposed group in the first 5 years of life in height was −1.8 cm (−3.5 to 0.2) and −1.7 cm (−3.2 to −0.2), and in weight was −1.6 kg (−3.2 to 0.12) and −0.9 kg (−2.6 to 0.9); compared with matched and post-famine controls. AD in the waist-to-hip ratio and waist-to-height ratio was 0.02 (0.01 to 0.03) and 0.01 (−0.01 to 0.02) and 0.01 (−0.0003 to 0.02) and 0.01 (−0.003 to 0.02) compared with matched and post-famine controls, respectively. AD in triceps skinfold was 1.4 mm (0.2 to 2.6) and 1.3 mm (0.1 to 2.5) in the exposed compared with matched and post-famine, respectively. AD in DBP was −2.4 mm Hg (−4.4 to −0.3) and 1.3 mm Hg (−0.8 to 3.4) in the exposed compared with matched and post-famine controls. Further details are given in figure 3 and online supplemental table 3.

AD in height in those exposed between 5 and 18 years was −0.9 cm (−2.5 to 0.7) and −3.5 cm (−6.3 to –0.7), and AD in weight was −0.7 kg (−2.9 to 1.4) and −1.0 kg (−4.6 to 2.6) compared with matched and post-famine controls, respectively. AD in DBP was −4.0 mm Hg (−6.7 to –1.2) and 5.1 mm Hg (0.9 to 9.3) and triceps skinfold thickness was 2.4 mm (0.9 to 3.9) and 2.8 mm (0.5 to 5.2) and handgrip strength was −2.2 kg (−3.9 to –0.4) and −2.2 kg (−3.9 to –0.4) in the exposed group compared with matched and post-famine controls, respectively (online supplemental table 4). The effect of exposure at the different chronological ages on anthropometry and NCD risk is presented in online supplemental table 5. In summary, when comparing risk by timing of exposure, individuals exposed during early childhood (0–2 years), preschool age (2–5 years) and late childhood (5–10 years) had reduced adult stature of −2.8 cm (−4.8 to –0.9); −2.8 cm (−4.7 to 0.9) and −2.1 cm (−4.0 to –0.2), and increased triceps skinfold thickness of 1.7 mm (−0.5 to 3.8), 3.2 (0.8 to 5.6) and 3.8 mm (1.6 to 6.0), respectively.

AD in the exposed group for arterial stiffness (higher values, except in pulse wave return time and ejection duration of the left ventricle, indicating stiffness) in brachial augmentation index was 5.4% (95% CI 0.3% to 10.5%) and 16.1% (95% CI 10.1% to 22.1%) compared with matched and post-famine controls, respectively, and in aortic augmentation index was 6.0% (95% CI 1.5% to 10.4%) and 11.7% (95% CI 6.1% to 17.3%) respectively. Additionally, AD in mean arterial pressure in the exposed group was −4.0 mm Hg (−6.1 to –1.9) and 1.8 (−0.5 to 4.1) and for pulse wave return time (lower value indicating stiffness) was 1.9 (−2.0 to 5.8) and −8.7 ms (−13.2 to –4.2) compared with the matched and post-famine controls, respectively.

AD in the first 1000 days of life in aortic augmentation index was 11.3% (2.1% to 20.5%) and 12.2% (4.2% to 20.2%) compared with matched and post-famine controls, respectively. AD in the mean arterial pressure was −3.4 mm Hg (−6.4 to –0.5) and 0.8 (–1.9 to 3.5) and for the pulse wave return time AD was 1.7 ms (−3.9 to 7.4) and −5.3 ms (–10.5 to –0.02) compared with matched and post-famine controls, respectively. Similar patterns of associations were observed when comparing arterial stiffness in those exposed during the first 5 years of age to their matched and post-famine controls. Detailed results for arterial stiffness are presented in table 2.

**Table 2:** Differences in arterial stiffness in famine-exposed versus matched and post-famine control groups in the whole sample, in the first 1000 days, and in the first 5 years

Outcomes	All exposed (n=528) compared with all matched controls (n=280)			All exposed (n=528) compared with all post-famine controls (n=158)		
	Model 1	Model 2	Model 3	Model 1	Model 2	Model 3
	β (95% CI); p value	β (95% CI); p value	β (95% CI); p value	β (95% CI); p value	β (95% CI); p value	β (95% CI); p value
Brachial augmentation index (Aix brachial) (%)	4.4 (−0.3 to 9.0); 0.06	4.1 (−0.3 to 8.4); 0.07	5.4 (0.3 to 10.5); 0.03*	22.0 (16.5 to 27.5); 0.01*	16.3 (10.5 to 22.2); 0.01*	16.1 (10.1 to 22.1); 0.01*
Aortic augmentation index (Aix aortic) (%)	3.1 (−0.8 to 7.1); 0.12	3.1 (−0.8 to 6.9); 0.12	6.0 (1.5 to 10.4); 0.01*	12.6 (7.7 to 17.6); 0.01*	11.4 (5.9 to 16.8); 0.01*	11.7 (6.1 to 17.3); 0.01*
Ejection duration of the left ventricle (ms)	−0.4 (−5.9 to 5.2); 0.90	−0.3 (−5.9 to 5.2); 0.91	−6.7 (−13.1 to –0.3); 0.04*	−1.9 (−8.5 to 4.7); 0.57	−0.9 (−8.2 to 6.5); 0.82	−2.0 (−9.5 to 5.6); 0.61
Pulse wave return time (ms)	4.5 (0.8 to 8.3); 0.02*	4.8 (1.4 to 8.2); 0.01*	1.9 (−2.0 to 5.8); 0.35	−13.3 (−17.8 to –8.8); 0.01*	−8.5 (−12.9 to –4.1) 0.01*	−8.7 (−13.2 to –4.2); 0.01*
Aortic pulse wave velocity (m/s)	−0.3 (−2.7 to 2.0); 0.78	−0.4 (−2.7 to 2.0); 0.77	−0.9 (−3.6 to 1.9); 0.53	2.7 (0.1 to 5.3); 0.04*	2.4 (−0.5 to 5.3); 0.11	2.8 (−0.1 to 5.7); 0.06
Central pulse pressure (mm Hg)	0.7 (−2.0 to 3.4); 0.61	0.6 (−2.1 to 3.3); 0.66	0.5 (−2.6 to 3.6); 0.74	2.9 (−0.9 to 6.6); 0.14	0.2 (−3.9 to 4.4); 0.92	0.2 (−4.1 to 4.4); 0.94
Central systolic blood pressure (mm Hg)	−4.2 (−13.3 to 5.0); 0.38	−4.2 (−13.4 to 4.9); 0.37	−0.5 (−11.2 to 10.2); 0.92	0.5 (−13.2 to 14.3); 0.94	−0.7 (−16.0 to 14.6); 0.93	−1.8 (−17.5 to 14.0); 0.83
Mean arterial pressure (mm Hg)	−3.6 (−5.4 to –1.8); 0.01*	−3.6 (−5.4 to –1.8); 0.01*	−4.0 (−6.1 to –1.9); 0.01*	2.7 (0.6 to 4.7); 0.01*	1.8 (−0.5 to 4.0); 0.12	1.8 (−0.5 to 4.1); 0.12
Brachial pulse pressure (mm Hg)	0.8 (−0.8 to 2.3); 0.33	0.8 (−0.8 to 2.3); 0.35	−0.02 (−1.8 to 1.8); 0.98	−0.7 (−2.6 to 1.2); 0.48	−1.5 (−3.6 to 0.7); 0.18	−1.6 (−3.8 to 0.6); 0.15
Heart rate	−0.6 (−2.2 to 1.0); 0.48	−0.6 (−2.2 to 1.0); 0.46	−0.2 (−2.0 to 1.7); 0.86	−0.8 (−2.9 to 1.2); 0.44	−0.7 (−2.9 to 1.5); 0.53	−0.8 (−3.1 to 1.4); 0.46
	Exposed in the 1000 days of life (n=214) compared with matched controls (n=110)			Exposed in the 1000 days of life (n=214) compared with post-famine controls (n=158)		
Brachial augmentation index (%)	1.5 (−5.1 to 8.0); 0.66	1.5 (−4.9 to 7.8); 0.65	4.2 (−3.3 to 11.7); 0.27	14.8 (9.0 to 20.7); 0.01*	12.6 (6.1 to 19.2); 0.01*	12.0 (5.3 to 18.7); 0.01*
Aortic augmentation index (%)	3.5 (−4.4 to 11.3); 0.38	3.5 (−4.3 to 11.3); 0.38	11.3 (2.1 to 20.5); 0.02*	11.0 (4.2 to 17.9); 0.01*	11.8 (4.1 to 19.6); 0.01*	12.2 (4.2 to 20.2); 0.01*
Ejection duration of the left ventricle (ms)	2.4 (−5.0 to 9.7); 0.53	2.3 (−5.0 to 9.7); 0.53	−3.2 (−11.9 to 5.5); 0.47	0.1 (−6.2 to 6.3); 0.98	2.8 (−4.3 to 9.9); 0.43	2.2 (−5.2 to 9.6); 0.56
Pulse wave return time (ms)	5.6 (0.1 to 11.0); 0.05*	5.6 (0.8 to 10.4); 0.02*	1.7 (−3.9 to 7.4; 0.55)	−8.8 (−13.7 to –3.8); 0.01*	−5.4 (−10.9 to –0.4); 0.04*	−5.3 (−10.5 to –0.02); 0.04*
Aortic pulse wave velocity (m/s)	−0.8 (−4.4 to 2.9); 0.69	−0.8 (−4.4 to 2.9); 0.68	−2.2 (−6.4 to 2.1); 0.32	2.0 (−2.0 to 4.9); 0.18	2.8 (−0.4 to 6.1); 0.09	2.6 (−0.7 to 6.0); 0.13
Central pulse pressure (mm Hg)	−3.4 (−7.9 to 1.1); 0.14	−3.4 (−7.9 to 1.1); 0.14	−2.8 (−8.1 to 2.6); 0.31	−1.0 (−5.8 to 3.9); 0.70	−2.0 (−7.5 to 3.5); 0.48	−2.7 (−8.4 to 2.9); 0.34
Central systolic blood pressure (mm Hg)	1.4 (−14.5 to 17.3); 0.87	1.4 (−14.4 to 17.3); 0.86	10.8 (−8.1 to 29.7; 0.26)	−0.2 (−20.9 to 20.5); 0.98	−7.4 (−30.8 to 16.1); 0.54	−6.4 (−30.9 to 18.1); 0.61
Mean arterial pressure (mm Hg)	−3.6 (−6.1 to –1.1); 0.01*	−3.6 (−6.1 to –1.1); 0.01*	−3.4 (−6.4 to –0.5); 0.02*	1.2 (−1.1 to 3.5); 0.29	1.0 (−1.6 to 3.6); 0.46	0.8 (−1.9 to 3.5); 0.54
Brachial pulse pressure (mm Hg)	0.1 (−2.1 to 2.5); 0.89	0.1 (−2.1 to 2.3); 0.90	−0.7 (−3.3 to 1.9); 0.60	−1.6 (−3.6 to 0.5); 0.14	−2.0 (−4.4 to 0.3); 0.09	−2.4 (−4.8 to –0.04); 0.04*
Heart rate (beat/minute)	0.1 (−2.4 to 2.6); 0.92	0.1 (−2.3 to 2.5); 0.92	0.2 (−2.7 to 3.1); 0.91	−0.03 (−2.5 to 2.4); 0.98	−1.4 (−4.0 to 1.1); 0.27	−1.5 (−4.3 to 1.2); 0.26
	Exposed in the first 5 years of life (n=321) compared with matched controls (n=174)			Exposed in the first 5 years of life (n=321) compared with post-famine controls (n=158)		
Aix brachial—brachial augmentation index (%)	3.1 (−2.7 to 8.8); 0.29	3.3 (−2.2 to 8.8); 0.24	4.2 (−2.2 to 10.6); 0.20	17.8 (12.1 to 23.4); 0.01*	12.4 (6.0 to 18.7); 0.01*	11.8 (5.2 to 18.3); 0.01*
Aix aortic—central augmentation index (%)	3.8 (−2.1 to 9.7); 0.21	3.9 (−2.0 to 9.8); 0.19	8.3 (1.5 to 15.1); 0.02*	11.7 (5.7 to 17.7); 0.01*	11.1 (4.2 to 18.1); 0.01*	11.4 (4.3 to 18.5); 0.01*
Ejection duration of the left ventricle (ms)	−0.6 (−7.7 to 6.4); 0.87	−0.7 (−7.7 to 6.4); 0.85	−4.9 (−13.0 to 3.3); 0.24	−2.0 (−8.7 to 4.8); 0.57	1.9 (−5.9 to 9.7); 0.63	1.7 (−6.4 to 9.7); 0.69
Pulse wave return time (ms)	7.1 (2.4 to 11.7); 0.01*	6.8 (2.7 to 10.9); 0.01*	3.4 (−1.3 to 8.2); 0.16	−10.0 (−14.7 to –5.3); 0.01*	−6.2 (−11.1 to –1.3); 0.01*	−5.8 (−10.9 to –0.8); 0.02*
Aortic pulse wave velocity (m/s)	−1.3 (−4.6 to 2.0); 0.43	−1.3 (−4.5 to 2.0); 0.45	−2.8 (−6.5 to 1.0); 0.15	2.4 (−0.4 to 5.2); 0.10	2.3 (−1.0 to 5.5); 0.17	2.0 (−1.3 to 5.4); 0.23
Central pulse pressure (mm Hg)	−1.1 (−4.8 to 2.4); 0.57	−1.0 (−4.6 to 2.7); 0.61	−1.3 (−5.5 to 3.0); 0.56	0.9 (−3.5 to 5.3); 0.68	−2.3 (−7.3 to 2.7); 0.37	−3.0 (−8.1 to 2.1); 0.25
Central systolic blood pressure (mm Hg)	−5.1 (−19.7 to 9.6); 0.50	−4.8 (−19.4 to 9.8); 0.52	1.7 (−15.3 to 18.8); 0.84	0.7 (−16.9 to 18.2); 0.94	−4.4 (−24.8 to 15.9); 0.67	−4.0 (−25.1 to 17.1); 0.71
Mean arterial pressure (mm Hg)	−3.0 (−5.3 to –0.7); 0.01*	−3.0 (−5.2 to –0.7); 0.01*	−2.9 (−5.5 to –0.2); 0.03*	2.6 (0.4 to 4.8); 0.02*	0.7 (−1.9 to 3.2); 0.60	0.7 (−2.0 to 3.3); 0.63
Brachial pulse pressure (mm Hg)	0.2 (−1.7 to 2.1); 0.83	0.2 (−1.7 to 2.1); 0.82	−0.7 (−2.9 to 1.5); 0.53	−1.3 (−3.2 to 0.6); 0.19	−2.0 (−4.2 to 0.3); 0.09	−2.3 (−4.6 to 0.02); 0.05
Heart rate	−1.0 (−3.1 to 1.2); 0.38	−0.9 (−3.0 to 1.2); 0.39	−0.3 (−2.7 to 2.1); 0.80	−0.6 (−2.9 to 1.6); 0.58	−0.7 (−3.2 to 1.8); 0.58	−1.1 (−3.7 to 1.5); 0.40

Model 1: unadjusted.

Model 2: adjusted for age and sex.

Model 3: model 2 further adjusted for religion, wealth status, educational status, physical activity, diet quality, life time alcohol and khat use.

* Statistically significant.

### Discussion

Famine survivors show several markers of poorer adult health and NCD risk compared with both matched and post-famine controls: shorter stature, lower weight, lower handgrip strength, higher waist-to-height ratio, higher triceps and subscapular skinfolds and greater arterial stiffness (suggesting raised cardiovascular risk). When compared with age-matched controls, famine survivors showed greater arterial stiffness but lower DBP. Conversely, when compared with post-famine controls, differences in arterial stiffness and DBP were concordant with higher values among famine survivors. No differences were found in sitting height, BMI, body fat, visceral fat, skeletal muscle mass or lifetime episodes of NCD. Associations were robust to adjustment for several factors known to increase NCD risk. In contrast to exposure in utero, risk was higher among those exposed during preschool and late childhood, likely due to prolonged exposure to famine or insufficient nutritional support.

The lower DBP observed among famine-exposed compared with matched controls and the higher DBP compared with the post-famine control is an unexpected and unique finding. This extends our previous Ethiopia study, which showed higher DBP in the 1980s famine survivors compared with born post-famine controls.^30 31^ A similar pattern was found in an age-matched and sex-matched cohort in the Democratic Republic of the Congo where survivors of childhood malnutrition had lower adult DBP.^32^ This observation could reflect the use of more robust control groups, given the limitations of using post-famine (thus younger) controls in many other studies. It might reflect a different prefamine phenotype risk (homeostatic capacity) in the exposed vs controls as well as small differences in diet and lifestyle which persist in the migrant community (ie*,* a lower metabolic load).^22^ It might also reflect too short a follow-up period. The observed increase in arterial stiffness is an important subclinical finding which suggests future clinical risk as DBP rises. Comparing concordance between BP and arterial stiffness in other settings may offer valuable insights.

We found that famine exposure was linked to higher waist-to-height ratio and subscapular skinfold thickness and triceps skinfold thickness, which may contribute to cardiometabolic disturbance, metabolic syndrome,^33 34^ abdominal obesity, insulin resistance, hyperglycaemia, hypertriglyceridaemia and type 2 diabetes.^35 36^ Lower handgrip strength in our study aligns with previous research on malnutrition survivors.^37^ It is a good marker of compromised muscle quality and a strong predictor of metabolic dysregulation and insulin resistance,^38^,^41^ poor general health outcomes, early all-cause and cardiovascular mortality and disability,^42 43^ and increased risk of early cognitive decline and poor functional outcomes.^44 45^ Age-related increases in arterial stiffness may further raise the risk of chronic NCDs such as cardiovascular disease, hypertension, coronary artery disease, stroke, heart failure, cognitive decline and increased mortality.^46 47^

Similar to our findings, studies on the Chinese famine indicated higher risk of NCD among those exposed before 18 years of age compared with post-famine controls.^20 48^ In contrast to our study (which found greater risk in those exposed during 2–10 years of age), the Dutch famine study linked increased risk of NCDs mainly with fetal exposure.^14 49 50^ Leningrad siege survivors who were exposed during their early life showed reduced height, weight and BMI and higher high-lipoprotein but showed no association with cardiovascular disease or target organ damage,^51^ and those exposed as adolescents showed worse outcomes than those exposed during early life.^52^ Numerous intrinsic and extrinsic factors before and after famine exposure may contribute to the observed associations. According to the thrifty phenotype hypothesis,^21^ early-life undernutrition induces physiological adjustments that promote short-term survival at a cost of increased NCD risk in subsequent resource-rich environments. The capacity-load model^22^ explains NCD as a mismatch between the capacity for homeostasis that develops in early life and the effects of adult lifestyle on the body. Early undernutrition may restrict metabolic capacity (physical growth and organ development) which, when combined with sedentary lifestyle and obesity (metabolic load), raises NCD risk. However, in agrarian societies with long-term food insecurity, limited access to animal-source high-fat diets and an active lifestyle, NCD risk might be lower. The impact of famine may also depend on its severity, duration, timing and the adequacy of subsequent relief.

Thus, our findings align with both the capacity load model and thrifty phenotype hypothesis. NCD risk depends on both the severity of early life undernutrition and later life factors that may worsen or mitigate this risk.^21 22^ This is vital for policy-makers to note. At present, metabolic load is relatively low in famine survivors, reflected in only moderate differences in adiposity; however, Ethiopia’s ongoing nutritional and economic transition, marked by sedentary lifestyles and poor-quality diets, may increase NCD risk. Effective preventive strategies include reducing intake of saturated fat, salt, sugar,^53^,^55^ alcohol and nicotine,^56 57^ promoting diets rich in fruits, grains, seeds and low-fat animal products,^58 59^ and increasing physical activity.^60 61^

There are several plausible explanations for the finding that famine survivors showed no significant differences in BMI, body composition or lifetime episodes of NCDs compared with the control groups. First, the overall cohort may be too young to manifest clinically overt NCDs. Second, partial recovery or catch-up growth among famine survivors may have diluted the differences. Third, lifestyle convergence between exposed and control groups, such as similar physical activity and environmental exposure, could have diluted variation. Lastly, a healthy survivor bias may also explain the absence of group differences. Nonetheless, the higher waist-to-height ratios and skinfold thickness observed among famine survivors suggest greater central rather than overall adiposity, a pattern consistent with evolutionary life history theory, which proposes that early undernutrition may favour an increased central fat deposition to enhance the capacity to resist infections.^62 63^ The increased arterial stiffness observed in famine survivors further supports this interpretation, indicating possible subclinical or ‘hidden’ metabolic vulnerability despite comparable BMI, fat and skeletal mass. Therefore, reliance on BMI, total body fat or skeletal mass alone may underestimate metabolic risk in famine survivors, underscoring the importance of assessing fat distribution and vascular health when evaluating the long-term consequences of early-life famine.

Strengths of our work include the novel use of two control groups, each with advantages and disadvantages, most importantly highlighting the limitation of post-famine controls which many other studies used. Post-famine controls are younger and inherently at lower NCD risk, potentially leading to false positive inferences. Our measurement of arterial stiffness as a subclinical marker of cardiovascular disease is also novel. Finally, our finding that NCD risk was greatest among those exposed in childhood has important implications for our understanding of the life-course aetiology of NCDs in low- and middle-income countries, and for policy makers. Although fetal life and infancy are considered key periods of sensitivity to environmental insults, the fetus and infant are partly buffered from direct exposure to malnutrition by maternal phenotype,^64^ whereas children lack this protection. We call for future research to include such early markers to better capture long-term risk and improve understanding of mechanisms of how exactly early-life famine/malnutrition leads to long-term NCD.

Limitations of our study include: exposure status relies on verbal report preventing precise assessment of famine severity. Control selection was based on screening to exclude individuals with known exposure to famine, drought or severe childhood malnutrition, without considering household food insecurity, which may not have fully captured all forms of early-life nutritional adversity. Although birth dates are self-reported and may be imprecise, such inaccuracies are likely to be comparable across exposed and control groups, and categorising age helps mitigate the resulting bias. We lack data on nutritional or rehabilitative support during survivor relocation. Relying on adult participants may introduce healthy survivor bias, as those most severely affected may not have survived. As a result, the cohort may include only the least affected individuals, reducing the difference between groups. Long-term health outcomes may require larger sample sizes to increase statistical power. Finally, we lack records of treatments provided which may have influenced long-term risk. Some evidence suggests that rapid post-malnutrition weight gain following nutritional treatment for malnutrition could exacerbate NCD risk.^65 66^

Implications: The findings have several direct implications for nutrition programming and NCD prevention in low- and middle-income settings. First, interventions should focus on ensuring food security and optimal treatment of childhood malnutrition to prevent short-term morbidity and mortality as well as the long-term metabolic consequences. Second, health systems in famine-affected areas should integrate routine screening for subclinical NCD markers, such as central adiposity and arterial stiffness, alongside traditional anthropometry. Third, NCD prevention strategies such as the promotion of healthy diets rich in fruits, grains, seeds and low-fat animal products, reduction of saturated fats, sugars and salt, increased physical activity and limiting alcohol and nicotine exposure should be prioritised for everyone, and, most importantly, for vulnerable populations with early-life nutritional insults. Finally, long-term routine follow-up and monitoring are essential to identify emerging NCD risk as nutritional transitions occur and lifestyles become more sedentary. Generally, the findings of this study emphasise the need for a dual approach in low- and middle-income settings: tackling immediate undernutrition while anticipating and mitigating long-term NCD risk in populations exposed to early-life severe malnutrition or famine. Overall, this study adds important insight at a time when famine and malnutrition risk is rising, and we hope it encourages future research into the biological mechanisms linking early-life malnutrition to later health and disease outcomes.

### Conclusion and recommendation

Early life famine survivors achieved smaller size and increased risk of several, but not all NCD markers compared with controls. Those exposed in childhood had the greatest risks. Discordant BP and arterial stiffness highlight the need to consider subclinical markers of NCD risk and conduct longer-term follow-up studies to better understand mechanistic issues. The findings underscores the need for ensuring food security, and prevention and optimal treatment of severe childhood malnutrition. Moreover, it highlights the need for the health systems in drought- and famine-affected regions to address elevated NCD risk in this vulnerable group by promoting healthy diets, active lifestyles and proactive NCD prevention, screening and management.

## Supplementary material

10.1136/bmjgh-2025-021721 online supplemental file 1

10.1136/bmjgh-2025-021721 online supplemental file 2

## Data Availability

Data are available on reasonable request.

## References

[1] Pizzorni M, Innocenti A, Tollin N (2024). Droughts and floods in a changing climate and implications for multi-hazard urban planning: A review. *City and Environment Interactions*.

[2] Tefera FF (2024). Ethiopia’s 1984/85 famine and the Red Terror Trials. Third World Q.

[3] The 1983-1985 Ethiopia drought/famine: disaster care report.

[4] Food security information network (FSIN) & global network against food crises (GNAFC) (2025). Global report on food crisis. https://www.fightfoodcrises.net/global-report-food-crises.

[5] Brown ME, Grace K, Billing T (2021). Considering climate and conflict conditions together to improve interventions that prevent child acute malnutrition. Lancet Planet Health.

[6] Barker D (1986). INFANT MORTALITY, CHILDHOOD NUTRITION, AND ISCHAEMIC HEART DISEASE IN ENGLAND AND WALES. The Lancet.

[7] Barker DJP, Osmond C, Winter PD (1989). WEIGHT IN INFANCY AND DEATH FROM ISCHAEMIC HEART DISEASE. The Lancet.

[8] Barker DJP (2007). The origins of the developmental origins theory. J Intern Med.

[9] Dewey KG, Begum K (2011). Long-term consequences of stunting in early life. Matern Child Nutr.

[10] Huang C, Phillips MR, Zhang Y (2013). Malnutrition in early life and adult mental health: evidence from a natural experiment. Soc Sci Med.

[11] Grey K, Gonzales GB, Abera M (2021). Severe malnutrition or famine exposure in childhood and cardiometabolic non-communicable disease later in life: a systematic review. BMJ Glob Health.

[12] Victora CG, Adair L, Fall C (2008). Maternal and child undernutrition: consequences for adult health and human capital. The Lancet.

[13] Elias SG, Keinan-Boker L, Peeters PHM (2004). Long term consequences of the 1944-1945 Dutch famine on the insulin-like growth factor axis. Int J Cancer.

[14] Roseboom T, de Rooij S, Painter R (2006). The Dutch famine and its long-term consequences for adult health. Early Hum Dev.

[15] Kyle UG, Pichard C (2006). The Dutch Famine of 1944-1945: a pathophysiological model of long-term consequences of wasting disease. Curr Opin Clin Nutr Metab Care.

[16] Lay MJ, Norling J (2020). The Consequences of the 1959–1961 Chinese Famine for Educational Attainment. B E J Econom Anal Policy.

[17] Cheng M, Wang Z, Yu NN (2024). Long-term mental health cost of the Great Chinese Famine. Health Econ.

[18] Almond D, Edlund L, Li H (2007). Long-term effects of the 1959-1961 China famine: mainland China and Hong Kong. http://www.nber.org/papers/w13384.pdf.

[19] Gooch E (2017). Estimating the Long-Term Impact of the Great Chinese Famine (1959–61) on Modern China. World Dev.

[20] Cheng M, Sommet N, Kerac M (2023). Exposure to the 1959-1961 Chinese famine and risk of non-communicable diseases in later life: A life course perspective. *PLOS Glob Public Health*.

[21] Hales CN, Barker DJP (2001). The thrifty phenotype hypothesis. Br Med Bull.

[22] Wells JCK (2018). The capacity-load model of non-communicable disease risk: understanding the effects of child malnutrition, ethnicity and the social determinants of health. Eur J Clin Nutr.

[23] Degefa M, Dubale B, Bayouh F (2020). Validation of the PHQ-9 depression scale in Ethiopian cancer patients attending the oncology clinic at Tikur Anbessa specialized hospital. BMC Psychiatry.

[24] Manzar MD, Salahuddin M, Alghadir A (2021). Psychometric properties of the Generalized Anxiety Disorder-7 Scale in Ethiopian university students. Bull Menninger Clin.

[25] Craig CL, Marshall AL (2003). International Physical Activity Questionnaire: 12-Country Reliability and Validity. Med Sci Sports Exerc.

[26] Herforth AW, Ballard T, Rzepa A (2024). Development of the Diet Quality Questionnaire for Measurement of Dietary Diversity and Other Diet Quality Indicators. *Curr Dev Nutr*.

[27] Kato T (2014). Development of the Sleep Quality Questionnaire in healthy adults. J Health Psychol.

[28] World Health Organization (2008). Training course on child growth assessment.

[29] Circumference, WHO Waist, and Waist-Hip Ratio (2008). Report of a who expert consultation.

[30] Arage G, Belachew T, Hassen H (2020). Effects of prenatal exposure to the 1983-1985 Ethiopian great famine on the metabolic syndrome in adults: a historical cohort study. Br J Nutr.

[31] Arage G, Belachew T, Abera M (2020). Consequences of early life exposure to the 1983-1985 Ethiopian Great Famine on cognitive function in adults: a historical cohort study. BMJ Open.

[32] Mwene-Batu P, Lemogoum D, de le Hoye L (2021). Association between severe acute malnutrition during childhood and blood pressure during adulthood in the eastern Democratic Republic of the Congo: the Lwiro cohort study. BMC Public Health.

[33] Ashwell M, Mayhew L, Richardson J (2014). Waist-to-height ratio is more predictive of years of life lost than body mass index. PLoS One.

[34] Castanheira M, Chor D, Braga JU (2018). Predicting cardiometabolic disturbances from waist-to-height ratio: findings from the Brazilian Longitudinal Study of Adult Health (ELSA-Brasil) baseline. Public Health Nutr.

[35] Ruiz-Alejos A, Carrillo-Larco RM, Miranda JJ (2020). Skinfold thickness and the incidence of type 2 diabetes mellitus and hypertension: an analysis of the PERU MIGRANT study. Public Health Nutr.

[36] González-Torres S, Anaya-Esparza LM, Trigueros Del Valle GF (2023). Skinfold Thickness as a Cardiometabolic Risk Predictor in Sedentary and Active Adult Populations. J Pers Med.

[37] Kirolos A, Harawa PP, Chimowa T (2024). Long-term outcomes after severe childhood malnutrition in adolescents in Malawi (LOSCM): a prospective observational cohort study. Lancet Child Adolesc Health.

[38] Lee MJ, Khang AR, Yi D (2022). Low relative hand grip strength is associated with a higher risk for diabetes and impaired fasting glucose among the Korean population. PLoS One.

[39] d’Avila J da C, Moreira El Nabbout TG, Georges Moreira El Nabbout H (2024). Correlation between low handgrip strength and metabolic syndrome in older adults: a systematic review. Arch Endocrinol Metab.

[40] Åström MJ, von Bonsdorff MB, Salonen MK (2021). Glucose regulation and grip strength in adults: Findings from the Helsinki Birth Cohort Study. Arch Gerontol Geriatr.

[41] Barbat-Artigas S, Rolland Y, Zamboni M (2012). How to assess functional status: A new muscle quality index. The Journal of Nutrition, Health and Aging.

[42] Soysal P, Hurst C, Demurtas J (2021). Handgrip strength and health outcomes: Umbrella review of systematic reviews with meta-analyses of observational studies. J Sport Health Sci.

[43] Vaishya R, Misra A, Vaish A (2024). Hand grip strength as a proposed new vital sign of health: a narrative review of evidences. J Health Popul Nutr.

[44] Shaughnessy KA, Hackney KJ, Clark BC (2020). A Narrative Review of Handgrip Strength and Cognitive Functioning: Bringing a New Characteristic to Muscle Memory. J Alzheimers Dis.

[45] Morera Á, Calatayud J, Casaña J (2023). Handgrip strength and work limitations: A prospective cohort study of 70,820 adults aged 50 and older. Maturitas.

[46] Shirwany NA, Zou M (2010). Arterial stiffness: a brief review. Acta Pharmacol Sin.

[47] Iulita MF, Noriega de la Colina A, Girouard H (2018). Arterial stiffness, cognitive impairment and dementia: confounding factor or real risk?. J Neurochem.

[48] He X, Shi X, Pan D (2023). Secular trend of non-communicable chronic disease prevalence throughout the life span who endured Chinese Great Famine (1959-1961). BMC Public Health.

[49] Roseboom TJ, van der Meulen JH, Ravelli AC (2001). Effects of prenatal exposure to the Dutch famine on adult disease in later life: an overview. Mol Cell Endocrinol.

[50] Ramirez D, Haas SA (2022). Windows of Vulnerability: Consequences of Exposure Timing during the Dutch Hunger Winter. Popul Dev Rev.

[51] Rotar O, Moguchaia E, Boyarinova M (2015). Seventy years after the siege of Leningrad: does early life famine still affect cardiovascular risk and aging?. J Hypertens.

[52] Sparén P, Vågerö D, Shestov DB (2004). Long term mortality after severe starvation during the siege of Leningrad: prospective cohort study. BMJ.

[53] Budreviciute A, Damiati S, Sabir DK (2020). Management and Prevention Strategies for Non-communicable Diseases (NCDs) and Their Risk Factors. Front Public Health.

[54] Ruthsatz M, Candeias V (2020). Non-communicable disease prevention, nutrition and aging. Acta Bio Medica Atenei Parm.

[55] Schwingshackl L, Heseker H, Kiesswetter E (2022). Dietary fat and fatty foods in the prevention of non-communicable diseases: A review of the evidence. Trends in Food Science & Technology.

[56] Parry CD, Patra J, Rehm J (2011). Alcohol consumption and non-communicable diseases: epidemiology and policy implications. Addiction.

[57] Glantz S, Gonzalez M (2012). Effective tobacco control is key to rapid progress in reduction of non-communicable diseases. The Lancet.

[58] Stanek A, Grygiel-Górniak B, Brożyna-Tkaczyk K (2023). The Influence of Dietary Interventions on Arterial Stiffness in Overweight and Obese Subjects. Nutrients.

[59] Pase MP, Grima NA, Sarris J (2011). The effects of dietary and nutrient interventions on arterial stiffness: a systematic review. Am J Clin Nutr.

[60] Sacre JW, Jennings GLR, Kingwell BA (2014). Exercise and dietary influences on arterial stiffness in cardiometabolic disease. Hypertension.

[61] Saz-Lara A, Cavero-Redondo I, Álvarez-Bueno C (2021). The Acute Effect of Exercise on Arterial Stiffness in Healthy Subjects: A Meta-Analysis. J Clin Med.

[62] Wells JCK (2009). Ethnic variability in adiposity and cardiovascular risk: the variable disease selection hypothesis. Int J Epidemiol.

[63] Wells JCK, Cole TJ, Cortina-Borja M (2019). Low Maternal Capital Predicts Life History Trade-Offs in Daughters: Why Adverse Outcomes Cluster in Individuals. Front Public Health.

[64] Wells JCK (2010). Maternal capital and the metabolic ghetto: An evolutionary perspective on the transgenerational basis of health inequalities. Am J Hum Biol.

[65] Thompson DS, McKenzie K, Opondo C (2023). Faster rehabilitation weight gain during childhood is associated with risk of non-communicable disease in adult survivors of severe acute malnutrition. *PLOS Glob Public Health*.

[66] Lelijveld N, Cox S, Anujuo K (2023). Post-malnutrition growth and its associations with child survival and non-communicable disease risk: a secondary analysis of the Malawi “ChroSAM” cohort. Public Health Nutr.

