# Supplementary material for: Forty years later: adult health and non-communicable disease following the 1984–1985 Great Ethiopian Famine – a retrospective cohort study
Source: BMJ Glob Health. 2026 Feb 23;11(2):e021721. doi: 10.1136/bmjgh-2025-021721 (PMC12931544; doi:10.1136/bmjgh-2025-021721)
Supplement: online supplemental file 1 [file bmjgh-11-2-s001.doc]

**Supplementary Table 1. Associations of exposure to the great Ethiopian famine compared to matched and post-famine control with markers of NCD risk after 4**0 years

| **Outcomes** | All exposed combined (n=528) compared to all matched control (n=280) | | | All exposed compared (n=528) to post-famine control (n=280) | | | **All exposed combined (n=528) compared to post-famine born from exposed parent (n=104)** | | |
| --- | --- | --- | --- | --- | --- | --- | --- | --- | --- |
|  | **Model 1** | **Model 2** | **Model 3** | **Model 1** | **Model 2** | **Model 3** | **Model 1** | **Model 2** | **Model 3** |
|  | β (95% CI, P-value) | β (95% CI, P-value) | β (95% CI, P-value) | β (95% CI, P-value) | β (95% CI, P-value) | β (95% CI, P-value) | β (95% CI, P-value) | β (95% CI, P-value) | β (95% CI, P-value) |
| Height | -1.5 (-2.8, -0.3; 0.01)* | -1.5 (-2.4, -0.6; 0.01)* | -1.4 (-2.4, -0.3; 0.01)* | -2.2 (-3.7, -0.6; 0.01)* | -2.8 (-4.1, -1.5; 0.01)* | -2.4 (-3.7, -1.1; 0.01)* | -3.2 (-5.0, -1.4; 0.01)* | -3.5 (-4.9, -2.0; 0.01)* | -3.2 (-4.7, -1.7; 0.01)* |
| Weight | -0.8 (-2.0, 0.4; 0.20) | -0.8 (-1.9, 0.4; 0.0.20) | -1.4 (-2.7, -0.07; 0,04)* | -1.7 (-3.2, -0.2; 0.03)* | -1.7 (-3.3, -0.1; 0.04)* | -1.0 (-2.6, 0.6; 0.23) | -2.4 (-4.2, -0.6; 0.01)* | -2.2(-4.1, -0.4; 0.02)* | -1.4 (-3.2, 0.4; 0.13) |
| Body mass index | 0.3 (-0.2, 0.9; 0.25) | 0.3 (-0.2, 0.8; 0.25) | 0.1 (-0.5, 0.7; 0.78) | 0.1 (-0.5, 0.8; 0.66) | 0.2 (-0.5, 0.9; 0.58) | 0.3 (-0.4, 1.0; 0.38) | 0.1 (-.6, 0.9; 0.75) | 0.2 (-0.7, 1.0; 0.71) | 0.3 (-0.5, 1.1; 0.48) |
| Sitting height | 0.2 (-0.5, 1.0; 0.58) | 0.2 (-0.5, 0.9; 0.53) | 0.2 (-0.6, 0.9; 0.69) | -0.3 (-1.2, 0.7; 0.56) | -0.9 (-1.8, 0.5; 0.06) | -0.8 (-1.8, 0.1; 0.08) | -1.1 (-2.2, 0.01; 0.05) | -1.6 (-2.7, -0.5; 0.01)* | -1.5 (-2.6, -0.4; 0.01)* |
| Mid-upper arm circumference | 0.4 (-0.03, 0.8; 0.07) | 0.4 (-0.02, 0.8; 0.06) | 0.2 (-0.3, 0.6; 0.45) | 0.1 (-0.4, 0.6; 0.75) | 0.3 (-0.2, 0.8; 0.27) | 0.5 (-0.1, 1.0; 0.09) | -0.02 ( -0.6, 0.6; 0.95) | 0.2(-0.4, 0.8; 0.59) | 0.4 (-0.21, 1.0; 0.20) |
| Blood pressure |  |  |  |  |  |  |  |  |  |
| Systolic blood pressure | -3.4 (-5.6, -1.2;0.01)* | -3.5 (-5.7, -1.3; 0.01)* | -4.1 (-6.7, -1.5; 0.01)* | 1.7 (-0.7, 4.1; 0.16) | 0.2 (-2.5, 2.8; 0.89) | -0.1 (-2.8, 2.6; 0.95) | 0.8 (-2.0, 3.5; 0.60) | -0.8 (-3.8, 2.3; 0.62) | -1.0 (-4.1, 2.1; 0.53) |
| Diastolic blood pressure | -2.6 (-4.0, -1.2; 0.01)* | -2.6 (-4.0, -1.2; 0.01)* | -2.8 (-4.4, -1.1; 0.01)* | 3.3 (1.6, 4.9; 0.01)* | 2.9 (1.0, 4.7; 0.01)* | 2.8 (0.9, 4.7; 0.01)* | 3.6 (1.6, 5.5; 0.01)* | 3.1 (1.0, 5.2; 0.01)* | 2.9 (0.7, 5.0; 0.01)* |
| Hypertension (yes/no) logistic | 0.7 (0.5, 1.0; 0.052) | 0.7 (0.4, 1.0; 0.04)* | 0.5 (0.3, 0.8; 0.01)* | 2.4 (1.2, 4.9; 0.02)* | 2.1 (0.96, 4.5; 0.06) | 1.9 (0.8, 4.2; 0.14) | 2.9 (1.1, 7.3; 0.03)* | 2.4 (0.9, 6.5; 0.07) | 2.0 (0.7, 5.6; 0.17) |
| Body composition |  |  |  |  |  |  |  |  |  |
| Body fat mass % | 0.4 (-1.1, 1.9; 0.61) | 0.4 (-0.6, 1.5; 0.42) | -0.2 (-1.5, 1.0; 0.73) | -1.1 (-2.9, 0.7; 0.23) | -0.3 (-1.7, 1.1; 0.72) | 0.6 (-1.2, 1.6; 0.79) | -0.3 (-2.5, 1.8; 0.77) | 0.2 (-1.5, 1.8; 0.87) | 0.5 (-1.2, 2.1; 0.56) |
| Visceral fat% | 0.3 (-0.1, 0.7; 0.20) | 0.3 (-0.1, 0.7; 19) | -0.1 (-0.5, 0.4; 0.75) | 0.7 (0.2, 1.2; 0.01)* | 0.6 (-0.1, 1.0; 0.11) | 0.6 (0.1, 1.2; 0.02)* | 0.7 (0.1, 1.3; 0.03) | 0.4 (-.3, 1.02; 0.25) | 0.6 (-0.01, 1.3; 0.05) |
| Skeletal mass % | -0.4 (-1.4, 0.7; 0.50) | -0.04 (-0.9, 0.1; 0.16) | -0.4 (-1.0, 0.2; 0.22) | -0.4 (-1.7, 0.9; 0.54) | -0.4 (-1.1, 0.4; 0.33) | -0.5 (-13, 0.2; 0.17) | -1.1 (-2.6, 0.5; 0.18) | -0.7 (-1.5, 0.2; 0.12) | -0.7 (-1.6, 0.1; 0.10) |
| Waist circumference | 0.9 (-0.2, 2.1; 0.12) | 0.9 (-0.2, 2.0; 0.12) | 0.03 (-1.3, 1.3; 0.96) | 1.1 (-0.3, 2.5; 0.11) | 0.7 (-0.8, 2.2; 0.38) | 0.8 (-0.8, 2.3; 0.33) | 0.9 (-0.8, 2.5; 0.31) | 0.5 (-1.3, 2.3; 0.59) | 0.8 (-1.0, 2.5; 0.41) |
| Hip circumference | 0.2 (-0.7, 1.2; 0.61) | 0.2 (-0.7, 1.2; 0.62) | -0.8 (-1.9, 0.3; 0.16) | -0.6 (-1.8, 0.7; 0.37) | -0.5 (-1.8, 0.9; 0.49) | 0.02 (-1.3, 1.4; 0.97) | -0.7 (-2.2, 0.7; 0.31) | -0.7 ( -2.2, 0.9; 0.38) | 0.1 ( -1.5, 1.6; 0.94) |
| Waist/hip ratio | 0.01 (-0.002, 0.02; 0.12) | 0.01 (-0.002, 0.02; 0.10) | 0.01 (-0.003, 0.02; 0.14) | 0.02 (0.01, 0.03; 0.01)* | 0.01 (-0.0, 0.03; 0.05) | 0.01 (-0.004, 0.02; 0.19) | 0.02 (0.003, 0.03; 0.02)* | 0.01 (-0.001, 0.03; 0.08) | 0.01 (-0.01, 0.02; 0.20) |
| Waist/height ratio | 0.01 (0.003, 0.02; 0.01)* | 0.01 (0.003, 0.02; 0.01)* | 0.004 (-0.004, 0.01; 0.35) | 0.01 (0.001, 0.02; 0.01)* | 0.01 (0.001, 0.02; 0.02)* | 0.01 (0.001, 0.02; 0.03)* | 0.01 (0.003, 0.02; 0.01)* | 0.01 (0.001, 0.02; 0.03)* | 0.01 (0.002, 0.02; 0.02)* |
| Subscapular skinfold | 1.3 (0.4, 2.1; 0.01)* | 1.2 (0.5, 2.0; 0.01)* | 1.1 (0.2, 1.9; 0.02)* | 1.1 (0.05, 2.2; 0.04)* | 0.8 (-0.3, 1.9; 0.14) | 1.2 (0.1, 2.3; 0.04)* | 1.5 (0.3, 2.7; 0.02)* | 1.1 (-.12, 2.4; 0.08) | 1.4 (0.2, 2.6; 0.03)* |
| Triceps skinfold | 1.8 (0.9, 2.7; 0.01)* | 1.8 (1.0, 2.5; 0.01)* | 1.8 (0.8, 2.7; 0.001* | 1.8 (0.6, 3.0; 0.01)* | 1.8 (0.7, 3.0; 0.01)* | 2.1 (1.0, 3.3; 0.01)* | 2.2 (0.8, 3.6; 0.01)* | 2.1 (0.7, 3.4; 0.01)* | 2.3 (1.0, 3.7; 0.01)* |
| Handgrip strength | -2.3 (-3.4, -1.2; 0.01)* | -2.2 (-3.1, -1.3; 0.01)* | -1.9 (-2.9, -0.8; 0.01)* | -4.1 (-5.5, -2.7; 0.01)* | -3.0 (-4.3, -1.7; 0.01)* | -2.6 (-3.9, -1.3; 0.01)* | -5.2 (-6.8, -3.5; 0.01)* | -3.8 ( -5.3, -2.3; 0.01)* | -3.5 (-5.0, -2.1; 0.01)* |
| **Chronic illness, logistic** | COR (95% CI, P-value) | AOR (95% CI, P-value) | AOR (95% CI, P-value) | COR (95% CI, P-value) | AOR (95% CI, P-value) | AOR (95% CI, P-value) | COR (95% CI, P-value) | AOR (95% CI, P-value) | AOR (95% CI, P-value) |
| Current anxiety | 1.8 (0.9, 3.5; 0.09) | 1.7 (0.9, 3.4; 0.10) | 1.4 (0.6, 3.5; 0.49) | 4.1 (1.2, 12.4; 0.02)* | 2.6 (0.7, 9.0; 0.15) | 1.6 (0.4, 6.4; 0.48) | 2.7 (0.8, 8.8; 0.11) | 1.6 (0.5, 5.8; 0.45) | 0.9 (0.2, 3.6; 0.88) |
| Current depression | 1.3 (0.8, 2.0; 0.40) | 1.2 (0.8, 2.0; 0.40) | 1.4 (0.8, 2.5; 0.28) | 2.9 (1.3, 6.4; 0.01)* | 1.6 (0.6, 3.5; 3.40) | 1.1 (0.4, 2.8; 0.87) | 1.8 (0.8, 4.1; 0.14) | 0.9 (0.4, 2.2; 0.86) | 0.6 (0.2, 1 .7; 0.37) |
| Life time all noncommunicable disease (yes/no) | 0.9 (0.5, 1.4; 0.55) | 0.8 (0.5, 1.4; 0.45) | 0.8 (0.5, 1.5; 0.51) | 2.9 (1.1, 7.4; 0.03)* | 2.0 (0.7, 5.4; 0.19) | 2.0 (0.7, 5.6; 0.20) | 4.8 (1.2, 20.1; 0.03)* | 3.3 (0 .7, 14.3; 0.17) | 2.8 (0 .6, 12.3; 0.19) |

*: statistically significant

Model-1: unadjusted

Model-2: adjusted to age and sex

Model-3: model 2 further adjusted for religion, wealth status, educational status, physical activity, diet quality, life time alcohol and khat use

**Supplementary Table 2. Associations of exposure to the great Ethiopian famine in the first 1000 days of life compared to matched control and born post-famine control with markers of NCD risk after 4**0 years

| **Outcomes** | Exposed in the first 1000 days of life (n=214) compared to age matched control (110) | | | | Exposed in the first 1000 days of life (n=214) compared to born post-famine control (n=158) | | | |
| --- | --- | --- | --- | --- | --- | --- | --- | --- |
|  | **Model 1** | **Model 2** | **Model 3** | | **Model 1** | **Model 2** | **Model 3** | |
| **Anthropometry** | β (95% CI, P-value) | β (95% CI, P-value) | β (95% CI, P-value) | | β (95% CI, P-value) | β (95% CI, P-value) | β (95% CI, P-value) | |
| Height | -1.8 (-3.7, 0.1; 0.06) | -1.8 (-3.3, -0.4; 0.01)* | -1.5 (-3.2, 0.2;0.09) | | -2.7 (-4.5, -0.9; 0.01)* | -2.1 (-3.8, -0.5; 0.01)* | -1.6 (-3.2, 0.1; 0.06) | |
| Weight | -1.4 (-3.1, 0.4; 0.12) | -1.4 (-3.1, 0.3; 0.11) | -2.1 (-4.1, -0.1; 0.04)* | | -1.9 (-3.5, -0.2; 0.03)* | -1.6 (-3.4, 0.2; 0.08) | -0.9 (-2.7, 1.0; 0.36) | |
| Body mass index | 0.1 (-0.6, 0.7; 0.87) | 0.1 (-0.6, 0.7; 0.84) | -0.2 (-0.9, 0.6; 0.69) | | 0.1 (-0.5, 0.9; 0.72) | 0.2 (-0.5, 0.9; 0.62) | 0.4 (-0.3, 1.1; 0.30) | |
| Sitting height | 0.02 (-1.0, 1.1; 0.97) | 0.01 (-0.9, 0.9; 1.00) | -0.2 (-1.3, 0.9; 0.70) | | -0.8 (-1.7, 0.1; 0.10) | -0.6 (-1.4, 0.3; 0.21) | -0.5 (-1.4, 0.4; 0.24) | |
| Mid-upper arm circumference | 0.04 (-0.5, 0.6; 0.89) | 0.04 (-0.5, 0.6; 0.87) | -0.1 (-0.7, 0.6; 0.80) | | 0.1 (-0.4, 0.6; 0.75) | 0.1 (-0.5, 0.6; 0.78) | 0.2 (-0.4, 0.8; 0.51) | |
| Blood pressure |  |  |  | |  |  |  | |
| Systolic blood pressure | -0.9 (-3.7, 1.8; 0.51) | -0.9 (-3.7, 1.8; 0.50) | -1.3 (-4.5, 1.9 0.43) | | 0.01 (-2.6, 2.6; 0.99) | -0.3 (-3.2, 2.7; 0.86) | -1.0 (-4.0, 2.1; 0.54) | |
| Diastolic blood pressure | -2.0 (-3.9, -0.1; 0.04)* | -2.0 (-3.9, -0.1; 0.04)* | -1.8 (-4.0, 0.5; 0.13) | | 2.0 (0.2, 3.9; 0.03)* | 1.4 (-0.7, 3.4; 0.20) | 1.1 (-1.1, 3.3; 0.31) | |
| Hypertension (yes/no), logistic | 0.7 (0.4, 1.5; 0.41) | 0.7 (0.3, 1.5; 0.40) | 0.6 (0.2, 1.5; 0.29) | | 1.5 (0.7, 3.4; 0.32) | 0.9 (0.3, 2.4; 0.82) | 0.8 (0.3, 2.4; 0.75) | |
| Body composition | | | | | | | | |
| Body fat mass % | -0.7 (-3.0, 1.6; 0.56) | -0.5 (-2.1, 1.2; 0.57) | -1.3 (-3.3, 0.6; 0.18) | | -0.3 (-2.3, 1.8; 0.81) | -0.8 (-2.4, 0.8; 0.33) | -0.2 (-1.8, 1.4; 0.79) | |
| Visceral fat% | -0.1 (-0.6, 0.3; 0.57) | -0.1 (-0.5, 0.3; 0.61) | -0.4 (-0.9, 0.1; 0.12) | | 0.4 (0.02, 0.7; 0.04)* | 0.2 (-0.1, 0.6; 0.21) | 0.3 (-0.04, 0.7; 0.08) | |
| Skeletal mass % | 0.5 (-1.2, 2.1; 0.57) | 0.3 (-0.5, 1.1; 0.50) | 0.1 (-0.9, 1.1; 0.84) | | -0.5 (-2.0, 1.1; 57) | -0.02 (-0.9, 0.8; 95) | -0.2 (-1.1, 0.7; 62) | |
| Waist circumference | 0.8 (-0.8, 2.5; 0.32) | 0.8 (-0.8, 2.4; 0.33) | 0.3 (-1.6, 2.2; 0.78) | | 0.5 (-1.0, 1.9; 55) | 0.3 (-1.4, 2.0; 75) | 0.4 (-1.3, 2.1; 646) | |
| Hip circumference | -0.2 (-1.6, 1.2; 0.76) | -0.2 (-1.6, 1.2; 0.78) | -1.0 (-2.6, 0.7; 0.24) | | -0.5 (-1.8, 0.8; 0.48) | -0.2 (-1.7, 1.3; 0.80) | 0.2 (-1.3, 1.7; 0.77) | |
| Waist/hip ratio | 0.01 (-0.003, 0.03; 0.13) | 0.01 (-0.01, 0.02; 0.12) | 0.01 (-0.01, 0.03; 0.16) | | 0.01 (-0.003, 0.02; 0.12) | 0.01 (-0.009, 0.02; 0.50) | 0.01 (-0.01, 0.02; 0.78) | |
| Waist/height ratio | 0.01 (-0.0, 0.02; 0.05) | 0.01 (0.0, 0.02; 0.04)* | 0.01 (-0.01, 0.02; 0.36) | | 0.01 (0.0, 0.02; 0.04)* | 0.01 (-0.003, 0.02; 0.19) | 0.01 (-0.005, 0.02; 0.28) | |
| Subscapular skinfold | 0.2 (-1.0, 1.4; 0.73) | 0.2 (-0.9, 1.3; 0.70) | 0.2 (-1.3, 1.3; 0.98) | | 0.6 (-0.5, 1.8; 0.28) | 0.4 (-0.8, 1.7; 0.47) | 0.7 (-0.6, 1.9; 0.29) | |
| Triceps skinfold | 0.7 (-0.7, 2.2; 0.31) | 0.8 (-0.5, 2.0; 0.22) | 0.6 (-0.9, 2.0; 0.43) | | 1.5 (0.3, 2.8; 0.02)* | 1.1 (-0.2, 2.3; 0.09) | 1.1 (-0.2, 2.3; 0.09) | |
| Handgrip strength | -1.0 (-2.6, 0.6; 0.23) | -1.0 (-2.2, 0.3; 0.14) | -0.7 (-2.2, 0.8; 0.37) | | -3.0 (-4.6, -1.5; 0.01)* | -2.1 (-3.5, -0.7; 0.01)* | -1.8 (-3.2, -0.4; 0.01)* | |
| **Chronic illness, logistic** | COR (95% CI, P-value) | AOR (95% CI, P-value) | AOR (95% CI, P-value) | COR (95% CI, P-value) | | AOR (95% CI, P-value) | | AOR (95% CI, P-value) |
| Current anxiety | 1.9 (0.5, 7.1; 0.34) | 1.9 (0.5, 7.1; 0.33) | 0.6 (0.1, 3.5; 0.59) | | 2.4 (0.6, 8.8; 0.19) | 2.7 (0.7, 10.8; 0.16) | 2.6 (0.6, 11.7; 0.22) | |
| Current depression | 0.7 (0.3, 1.7; 0.43) | 0.7 (0.3, 1.7; 0.43) | 0.6 (0.2, 1.8; 0.33) | | 1.3 (0.5, 3.4; 0.57) | 1.5 (0.5, 4.2; 0.45) | 1.6 (0.5, 4.8; 0.40) | |
| Life time all non-communicable disease (yes/no) | 1.2 (0.5, 3.1; 0.68) | 1.2 (0.5, 3.1; 0.69) | 1.1 (0.4, 3.3; 0.88) | | 2.2 (0.8, 6.1; 0.14) | 1.2 (0.3, 4.1; 0.80) | 1.0 (0.3, 4.0; 0.95) | |

*: statistically significant

Model-1: unadjusted

Model-2: adjusted to age and sex

Model-3: model 2 further adjusted for religion, wealth status, educational status, physical activity, diet quality, life time alcohol and khat use

**Supplementary Table 3. Associations of exposure to the great Ethiopian famine in the first 5 years of life compared to matched controls and born post-famine control with markers of NCD risk after 4**0 years

| **Outcomes** | Exposure in the first 5 years of life (n=321) compared to their age matched control (n=174) | | | Exposure in the first 5-years of life (n=321) compared to born post-famine control (n=158) | | |
| --- | --- | --- | --- | --- | --- | --- |
|  | **Model 1** | **Model 2** | **Model 3** | **Model 1** | **Model 2** | **Model 3** |
| **Anthropometry** | β (95% CI, P-value) | β (95% CI, P-value) | β (95% CI, P-value) | β (95% CI, P-value) | β (95% CI, P-value) | β (95% CI, P-value) |
| Height | -1.7 (-3.3, -0.1; 0.03)* | -1.8 (-3.0, -0.6; 0.01)* | -1.8 (-3.5, -0.2;0.03)* | -2.5 (-4.2, -0.9; 0.01)* | -2.2 (-3.7, -0.7; 0.01)* | -1.7(-3.2, -0.2; 0.03)* |
| Weight | -1.1 (-2.6, 0.4; 0.14) | -1.2 (-2.6, 0.3; 0.12) | -1.6 (-3.2, 0.12; 0.07) | -1.7 (-3.3, -0.2; 0.03)* | -1.7 (-3.4, 0.1; 0.07) | -0.9 (-2.6, 0.9; 0.33) |
| Body mass index | 0.2 (-0.4, 0.9; 0.49) | 0.2 (-0.4, 0.9; 0.46) | 0.1 (-0.6, 0.9; 0.70) | 0.2 (-0.4, 0.9; 0.51) | 0.02 (-0.7, 0.8; 0.96) | 0.1 (-0.7, 0.9; 0.75) |
| Sitting height | 0.03 (-0.8, 0.9; 0.95) | -0.02 (-0.8, 0.7; 0.96) | -0.3 (-1.2, 0.6; 0.47) | -0.6 (-1.5, 0.2; 0.16) | -0.7 (-1.5, 0.2; 0.12) | -0.7 (-1.5, 0.2; 0.13) |
| Mid-upper arm circumference | 0.2 (-0.3, 0.7; 0.36) | 0.2 (-0.2, 0.7; 0.33) | 0.1 (-0.4, 0.7; 0.62) | 0.2 (-0.3, 0.7; 0.41) | 0.04 (-0.5, 0.6; 0.88) | 0.2 (-0.4, 0.7; 0.51) |
| Blood pressure | | | | | | |
| Systolic blood pressure | -2.4 (-5.2, 0.3; 0.08) | -2.5 (-5.2, 0.3; 0.08) | -3.0 (-6.1, 0.2; 0.07) | 0.9 (-1.7, 3.4; 0.49) | -0.2 (-3.2, 2.7; 0.87) | -0.6 (-3.6, 2.5; 0.71) |
| Diastolic blood pressure | -2.3 (-4.1, -0.6; 0.01)* | -2.3 (-4.1, -0.6; 0.01)* | -2.4 (-4.4, -0.3; 0.02)* | 2.9 (1.1, 4.7; 0.01)* | 1.5 (-0.6, 3.5; 0.16) | 1.3 (-0.8, 3.4; 0.23) |
| Hypertension (yes/no), logistic | 0.9 (0.5, 1.5; 0.65) | 0.9 (0.5, 1.5; 0.65) | 0.7 (0.3, 1.3; 0.22) | 2.2 (1.0, 4.7; 0.04)* | 1.2 (0.5, 2.9; 0.71) | 1.1 (0.4, 2.9; 0.82) |
| Body composition | | | | | | |
| Fat mass % | -0.7 (-2.7, 1.3; 0.48) | -0.3 (-1.7, 1.1; 0.66) | -1.0 (-2.5, 0.6; 0.23) | -0.8 (-2.8, 1.2; 0.43) | -0.5 (-2.0, 1.0; 0.52) | 0.02 (-1.5, 1.6; 0.98) |
| Visceral fat | -0.1 (-0.5, 0.3; 0.66) | -0.1 (-0.4, 0.3; 0.75) | -0.3 (-0.7, 0.2; 0.22) | 0.4 (0.1, 0.8; 0.02)* | 0.3 (-0.1, 0.7; 0.19) | 0.4 (0.01, 0.8; 0.04)* |
| Skeletal mass % | 0.3 (-1.2, 1.7; 0.73) | -0.1 (-0.8, 0.6; 0.80) | -0.3 (-1.1, 0.6; 0.52) | -0.2 (-1.6, 1.3; 0.81) | -0.2 (-1.0, 0.7; 0.69) | -0.4 (-1.2, 0.5; 0.42) |
| Waist circumference | 1.2 (-0.2, 2.6; 0.09) | 1.2 (-0.2, 2.6; 0.10) | 0.8 (-0.8, 2.4; 0.33) | 0.7 (-0.7, 2.1; 32) | 0.4 (-1.2, 2.0; 61) | 0.6 (-1.0, 2.2; 47) |
| Hip circumference | -0.1 (-1.3, 1.1; 0.86) | -0.08 (-1.3, 1.1; 0.89) | -1.1 (-2.5, 0.3; 0.11) | -0.7 (-1.9, 0.6; 0.31) | -0.3 (-1.7, 1.2; 0.73) | 0.3 (-1.2, 1.7; 0.73) |
| Waist/hip ratio | 0.02 (0.01, 0.03; 0.02)* | 0.02 (0.01, 0.03; 0.02)* | 0.02 (0.01, 0.03; 0.01)* | 0.02 (0.003, 0.03; 0.02)* | 0.01 (-0.01, 0.02; 0.30) | 0.01 (-0.01, 0.02; 0.55) |
| Waist/height ratio | 0.01 (0.01, 0.02; 0.01)* | 0.01 (0.01, 0.02; 0.01)* | 0.01 (-0.001, 0.02; 0.06) | 0.01 (0.01, 0.02; 0.02)* | 0.01 -(0.002, 0.02; 0.12) | 0.01 (-0.003, 0.02; 0.16) |
| Subscapular skinfold thickness | 0.8 (-0.3, 1.8; 0.15) | 0.8 (-0.1, 1.7; 0.10) | 0.6 (-0.5, 1.7; 0.271) | 1.0 (-0.1, 2.1; 0.10) | 0.4 (-0.8, 1.6; 0.53) | 0.7 (-0.5, 1.9; 0.28) |
| Triceps skinfold thickness | 1.5 (0.3, 2.7; 0.02)* | 1.5 (0.5, 2.6; 0.01)* | 1.4 (0.2, 2.6; 0.02)* | 1.8 (0.5, 3.0; 0.01*) | 1.2 (-0.02, 2.4; 0.05) | 1.3 (0.05, 2.5; 0.04)* |
| Handgrip strength | -1.7 (-3.1, -0.3; 0.01) * | -1.8 (-2.9, -0.7; 0.01)* | -1.7 (-2.9, -0.4; 0.01) * | -3.3 (-4.8, -1.8; 0.01)* | -2.4 (-3.8, -1.1; 0.01)* | -2.2 (-3.5, -0.8; 0.01) * |
| Chronic illness, logistic | COR (95% CI, P-value) | AOR (95% CI, P-value) | AOR (95% CI, P-value) | COR (95% CI, P-value) | AOR (95% CI, P-value) | AOR (95% CI, P-value) |
| Current anxiety | 2.4 (0.8, 7.2; 0.13) | 2.4 (0.8, 7.3; 0.12) | 0.9 (0.2, 3.7; 0.88) | 2.9 (0.8, 10.0; 0.10) | 2.9 (0.8, 11.0; 0.12) | 2.9 (0.6, 11.8; 0.17) |
| Current depression | 0.9 (0.4, 1.8; 0.715) | 0.9 (0.4, 1.8; 0.71) | 0.8 (0.3, 1.8; 0.55) | 1.7 (0.7, 4.0; 0.25) | 1.5 (0.5, 3.9; 0.46) | 1.5 (0.5, 4.5; 0.43) |
| Life time all noncommunicable disease (yes/no) | 1.5 (0.7, 3.5; 0.32) | 1.5 (0.7, 3.5; 0.32) | 1.4 (0.5, 3.6; 0.47) | 2.2 (0.8, 6.0; 0.11) | 1.5 (0.5, 4.8; 0.45) | 1.4 (0.4, 4.6; 0.55) |

*: statistically significant

Model-1: unadjusted

Model-2: adjusted for age and sex

Model-3: model 2 further adjusted for religion, wealth status, educational status, physical activity, diet quality, life time alcohol and khat use

**Supplementary Table 4. Associations of exposure to the great Ethiopian famine** between 5 and 18 years of life compared to matched controls and born post-famine control with markers of NCD risk after 40 years

| **Outcomes** | Exposure between 5 and 18 years of life (n=207)) compared to their age matched control (106) | | | Exposure between 5 and 18 years of life (n=207) compared to born post-famine control (n=158) | | |
| --- | --- | --- | --- | --- | --- | --- |
|  | **Model 1** | **Model 2** | **Model 3** | **Model 1** | **Model 2** | **Model 3** |
| **Anthropometry** | β (95% CI, P-value) | β (95% CI, P-value) | β (95% CI, P-value) | β (95% CI, P-value) | β (95% CI, P-value) | β (95% CI, P-value) |
| Height | -1.3(-3.2, 0.7; 0.20) | -1.1 (-2.4, 0.3; 0.14) | -0.9 (-2.5, 0.7; 0.28) | -1.5 (-3.4, 0.3; 0.11) | -3.5 (-6.3, -0.58; 0.02)* | -3.5 (-6.3, -.7; 0.02)* |
| Weight | -0.2(-2.2, 1.9; 0.87) | -0.02 (-1.9, 1.9; 0.98) | -0.7 (-2.9, 1.4; 0.51) | -1.6 ( -3.5, 0.3; 0.10) | -0.9 (-4.6, 2.9; 0.65) | -1.0 (-4.6, 2.6; 0.58) |
| Body mass index | 0.6(-0.3, 1.5; 0.18) | 0.6 (-0.3, 1.5; 0.19) | 0.4( -0.6, 1.5; 0.43) | 0.2 (-0.6, 1.0; 0.65) | 0.9 (-0.8, 2.5; 0.29) | 1.0 (-0.8, 2.5; 0.287) |
| Sitting height | 0.2(-0.6, 1.0; 0.58) | 0.6(-0.7, 1.8; 0.37) | 1.0 (-0.5, 2.4; 0.20) | 0.2 (-1.0, 1.4; 0.72) | -1.8 (-4.2, 0.5; 0.12) | -1.8 ( -4.1, 0 .5; 0.12 ) |
| Mid-upper arm circumference | 0.7(-0.01, 1.3; 0.05) | 0.6 (-0.02, 1.3; 0.06) | 0.3(-0.5, 1.0; 0.47) | -0.1(-0.7, 0.5; 0.70) | 0.9 (-0.3, 2.1; 0.16) | 1.0(-0.3, 2.07; 0.13) |
|  | | | | | | |
| Systolic blood pressure | -5.0 (-8.6, -1.3;0.01)* | -5.0(-8.6, -1.3; 0.01)* | -5.9 (-10.2, -1.6; 0.01)* | 2.7 (-0.1, 5.4; 0.05) | -2.5 (-8.3, 3.2; 0.39) | -3.5 (-9.4, 2.4; 0.25) |
| Diastolic blood pressure | -3.2(-5.5, -0.8;0.01)* | -3.2 (-5.5, -0.9; 0.01)* | -4.0 (-6.7, -1.2; 0.01)* | 3.6 (1.7, 5.6; 0.01)* | 5.2 (1.1, 9.3;0.01)* | 5.1 (0.9, 9.3; 0.02)* |
| Hypertension (yes/no), logistic | 0.5 (0.3, 1.0; 0.03)* | 0.5 (0.3, 0.9; 0.03)* | 0.3 (0.2, 0.7; 0.01)* | 2.4 (1.1, 5.3; 0.03)* | 4.8 (1.2, 18.9; 0.02)* | 3.2 (0.6, 16.7; 0.16) |
|  | | | | | | |
| Fat mass % | 1.8 (-0.5, 4.1; 0.13) | 1.5(-0.2, 3.2; 0.08) | 0.9(-1.12, 0.9; 0.37) | -1.8 (-3.8, 0.3; 0.09) | 0.4(-2.8, 3.6; 0.80) | 0.5(-2.7, 3.6; 0.77) |
| Visceral fat | 0.8 (-0.1, 1.6; 0.08) | 0.8 (-0.1, 1.6; 0.08) | 0.2(-0.8, 1.2; 0.71) | 1.1 (0.4, 1.8; 0.01)* | 1.7 (0.3, 3.2; 0.02)* | 1.8 (0.4, 3.3; 0.01)* |
| Skeletal mass % | -1.1 (-2.7, 0.5; 0.18) | -0.9 (-1.7, -0.13; 0.02)* | -0.6 (-1.5, 0.3; 0.18) | -0.7(-2.2, 0.8; 0.38) | -1.2(-2.8, 0.4; 0.15) | -1.2(-2.8, 0.4; 0.14) |
| Waist circumference | 0.6 (-1.3, 2.5; 0.54) | 0.7 (-1.2, 2.6; 0.49) | -0.7 (-2.9, 1.4; 0.49) | 1.7 (0.04, 3.4; 0.04)* | 2.2(-1.3, 5.7; 0.23) | 1.8 (-1.6, 5.3; 0.30) |
| Hip circumference | 0.8 (-0.8, 2.3; 0.35) | 0.7 (-0.8, 2.3; 0.36) | -0.2 (-1.9, 1.6; 0.87) | -0.5(-1.9, 0.9; 0.49) | -0.02 (-3.0, 3.0; 0.99) | 0.01(-2.9, 2.9; 1.0) |
| Waist/hip ratio | -0.001 (-0.02, 0.02; 0.93) | 0.0001(-0.02, 0.02; 1.0) | -0.01(-0.03, 0.01; 0.45) | 0.03 (0.01, 0.1; 0.01)* | 0.02 (-0.01, 0.05; 0.11) | 0.02 (-0.01, 0.1; 0.20) |
| Waist/height ratio | 0.01(-0.01, 0.02; 0.25) | 0.01 (-0.01, 0.02; 0.26) | -0.01(-0.02, 0.01; 0.65) | 0.01 (0.01, 0.01; 0.01)* | 0.02(0.001, 0.05; 0.04)* | 0.02 (-0.002, 0.04; 0.07) |
| Subscapular skinfold thickness | 2.0 (0.7, 3.3; 0.01)* | 1.8 (0.4, 3.2; 0.01)* | 1.8 (0.4, 3.2; 0.01)* | 1.3(0.1, 2.5; 0.04)* | 0.9 (-1.7, 3.4; 0.50) | 1.0 ( -1.4, 3.4; 0.41) |
| Triceps skinfold thickness | 2.4 (0.9, 3.8; 0.01)* | 2.3 (1.0, 3.6; 0.01)* | 2.4 (0.9, 3.9; 0.01)* | 1.7 (0.4, 3.0; 0.01)* | 2.8 (0.4, 5.3; 0.02)* | 2.8 (0.5, 5.2; 0.02)* |
| Handgrip strength | -3.0(-4.8, -1.1; 0.01)* | -2.9 (-4.4, -1.3; 0.01)* | -2.2 (-3.9, -0.4; 0.02)* | -5.2(-6.9, -3.5; 0.01)* | -3.3 (-6.2, -0.4; 0.03)* | -3.1 (-6.0, -0.2; 0.03)* |
| Chronic illness, logistic | COR (95% CI, P-value) | AOR (95% CI, P-value) | AOR (95% CI, P-value) | COR (95% CI, P-value) | AOR (95% CI, P-value) | AOR (95% CI, P-value) |
| Current anxiety | 2.1 (1.0, 4.4; 0.05) | 2.0 (1.0, 4.4; 0.06) | 2.4 (1.0, 5.8; 0.06) | 5.3 (1.5, 18.0; 0.01)* | 3.3 (0.5, 20.7; 0.21) | 0.4 (0.03, 5.8; 0.50) |
| Current depression | 2.2 (0.8, 5.8; 0.14) | 2.1 (0.8, 5.8; 0.14) | 2.0 (0.6, 7.1; 0.30) | 4.5 (1.9, 10.2; 0.01)* | 2.2 (0.6, 8.3; 0.26) | 0.8 (0.2, 4.0; 0.82) |
| Life time all noncommunicable disease; (yes/no) | 0.6(0.3, 1.1; 0.07) | 0.5 (0.3, 1.0; 0.07) | 0.6 (0.3, 1.3; 0.18) | 3.8 (1.4, 10.1; 0.01)* | 1.7 (0.3, 8.5; 0.53) | 1.8 (0.3, 9.8; 0.53) |

*: statistically significant

Model-1: unadjusted

Model-2: adjusted for age and sex

Model-3: model 2 further adjusted for religion, wealth status, educational status, physical activity, diet quality, life time alcohol and khat use

**Supplementary Table 5. Associations of exposure to the great Ethiopian famine at different developmental stage compared to their matched controls with risks of NCD after 4**0 years

| **Outcomes** | **All exposed compared to matched controls** | | | | | Post-famine born to exposed parent compared to post-famine born to non-exposed parents |
| --- | --- | --- | --- | --- | --- | --- |
|  | **Fetal exposure** | **Early childhood (0-2 yrs)** | **Preschool (2-5 yrs)** | **Late childhood (5-10 yrs)** | **Adolescence (10-18 yrs)** | Post-famine born to exposed parent |
|  | β (95% CI, P-value) | β (95% CI, P-value) | β (95% CI, P-value) | β (95% CI, P-value) | β (95% CI, P-value) | β (95% CI, P-value) |
| Height | -0.9(-2.9, 1.1; 0.36) | -2.8(-4.8, -0.9; 0.01) * | -2.8 (-4.7, -0.9; 0.01) * | -2.1(-4.0, -0.2; 0.03) * | 0.4(-1.7, 2.4; 0.74) | 0.2(-3.1, 3.5; 0.92) |
| Sitting height | -0.9(-2.9, 1.1; 0.36) | -2.9(-4.7, -0.9; 0.01) * | 0.4 (-1.7, 2.4; 0.74) | -0.3 (-1.4, 0.7; 0.52) | 0.4(-1.0, 1.7; 0.63) | 0.7(-3.3, 4.7; 0.73) |
| Weight | -2.0(-4.9, 1.0; 0.19) | -1.7(-4.3, 0.9; 0.19) | -2.1(-5.1, 0.8; 0.16) | 0.5 (-2.7, 3.7; 0.76) | -1.9(-4.7, 0.9; 0.19) | 0.2 (-1.0, 1.5; 0.71) |
| Body mass index | -0.18(-1.5, 1.1; 0.78) | -0.2(-1.01, 0.63; 0.65) | 0.7(-0.8, 2.1; 0.39) | 1.3 (-0.4, 3.0; 0.14) | -0.7(-2.0, 0.7; 0.34) | 1.1 (-0.6, 2.9; 0.21) |
| Mid-upper arm circumference | -0.5(-1.4, 0.4; 0.30) | 0.3 (-0.5, 1.5; 0.47) | 0.4 (-0.5, 1.4; 0.38) | 0.9(-0.2, 1.9; 0.11) | -0.3 (-1.4, 0.9; 0.64) | 0.4 (-0.8, 1.5; 0.51) |
| Blood pressure |  |  |  |  |  |  |
| Systolic blood pressure | 0.4(-4.2, 5.0; 0.87) | -1.9 (-6.2, 2.4; 0.38) | -6.6 (-13.2, 0.1; 0.05) | -8.3 (-14.3, -2.3; 0.01) * | -4.7 (-11.1, 1.7; 0.15) | 6.8(0.6, 12.9; 0.03) * |
| Diastolic blood pressure | -1.5 (-4.8, 1.8; 0.37) | -2.0 (-5.0, 1.0; 0.18) | -4.4 (-8.4, -0.4; 0.03) * | -5.1(-9.2, -1.1; 0.01) * | -3.8(-7.5, -0.08; 0.04) * | 4.9 (0.1, 9.8; 0.048) * |
| Hypertension (yes/no), logistics | 0.5 (0.1, 2.3; 0.45) | 0.7 (0.2, 2.5; 0.63) | 0.6 (0.2, 1.7; 0.34) | 0.2 (0.1, 0.8; 0.02) * | 0.3 (0.1, 0.8; 0.02) * | 1.9 (0.3, 12.3; 0.49) |
| Body composition |  |  |  |  |  |  |
| Body fat mass % | -1.5(-4.4, 1.4; 0.30) | -1.1 (-3.6, 1.4; 0.38) | -1.01(-3.5, 1.5; 0.43) | 1.5 (-1.4, 4.5; 0.30) | -0.1(-2.8, 2.6; 0.94) | 0.6 (-2.7, 3.9; 0.70) |
| Visceral fat% | -0.5 (-1.2, .3; 0.22) | -0.3(-0.9, 0.3; 0.37) | 0.1(-0.7, 0.8; 1.0) | 0.8(-0.98, 2.5; 0.39) | -0.5(-1.4, 0.5; 0.36) | 0.1 (-0.7, 0.9; 0.82) |
| Skeletal mass % | 0.2(-1.1, 1.6; 0.75) | 0.05 (-1.3, 1.4; 0.95) | -0.7(-2.1, 0.7; 0.30) | -1.1(-2.5, 0.4; 0.14) | -0.3(-1.4, 0.9; 0.65) | 0.2 (-1.7, 2.1; 0.86) |
| Waist circumference | -0.2(-2.8, 2.5; 0.88) | 0.6 (-1.9, 3.03; 0.65) | 1.7(-1.2, 4.5; 0.25) | 0.7(-2.4, 3.7; 0.66) | -2.3(-5.4, 0.7; 0.14) | 0.7(-3.0, 4.5; 0.70) |
| Hip circumference | -1.3(-3.6, 1.1; 0.29) | -0.4(-2.5, 1.7; 0.72) | -1.7(-4.3, 0.9; 0.20) | 0.7 (-1.8, 3.3; 0.56) | -1.3 (-3.9, 1.2; 0.31) | 1.0 (-2.2, 4.2; 0.53) |
| Waist/hip ratio | 0.01(-0.02, 0.03; 0.57) | 0.01(-0.01, 0.03; 0.42) | 0.04(0.01, 0.1; 0.01) * | -0.001(-0.03, 0.02; 0.97) | -0.01 (-0.04, 0.02; 0.40) | 0.01(-0.003, 0.02; 0.14) |
| Waist/height ratio | -0.0003(-0.02, 02; 0.97) | 0.01(-0.01, 0.02; 0.23) | 0.02(0.003, 0.03; 0.03) * | 0.01 (-0.01, 0.03; 0.42) | -0.02(-0.03, 0.004; 0.12) | 0.003 (-0.02, 0.03; 0.79) |
| Subscapular skinfold | -1.02(-2.7, 0.7; 0.23) | 1.0(-1.0, 2.8; 0.35) | 1.4(-0.5,3.3; 0.14) | 2.9 (1.0, 4.7; 0.01) * | 1.01 (-1.2, 3.2; 0.37) | -1.6 (-4.1, 0.9; 0.21) |
| Triceps skinfold | -0.8 (-2.6, 0.9, 0.36) | 1.7(-0.5, 3.8; 0.13) | 3.2(0.8, 5.6; 0.01) * | 3.8(1.6, 6.02; 0.01) * | 1.0(-1.1, 3.1; 0.34) | -0.6 (-2.7, 1.5; 0.55) |
| Handgrip strength | 1.6 (-0.4, 3.6; 0.12) | -2.2(-4.4, -0.1; 0.04) * | -5.0(-7.1, -2.8; 0.01) * | -1.2(-3.9, 1.5; 0.38) | -2.5(-4.9, -0.1; 0.04) * | 0.4 (-2.6, 3.4; 0.79) |
| Chronic illness (logistic) | COR (95% CI); P-value | AOR (95% CI); P-value | AOR (95% CI); P-value | COR (95% CI); P-value | AOR (95% CI); P-value | AOR (95% CI); P-value |
| Current anxiety | 2.2 (0.2, 30.3; 0.55) | 0.1 (0.001, 4.0; 0.20) | 2.3 (0.1, 41.6; 0.56) | 1.8 (0.1, 29.0; 0.68) | 2.3 (0.4, 14.7; 0.38) | Not applicable (NA) |
| Current depression | 1.4 (0.3, 7.1; 0.66) | 0.3 (0.1, 1.6; 0.16) | 0.9 (0.2, 3.5; 0.85) | 2.4 (0.6, 10.1; 0.23) | 2.9 (0.8, 11.0, 0.12) | 0.6 (0.04, 9.7; 0.72) |
| Life time all noncommunicable disease, (yes/no) | 1.3 (0.2, 9.2; 0.81) | 0.9 (0.2, 3.6; 0.85) | 3.5 (0.4, 32.9; 0.27) | 1.0 (0.3, 3.4; 1.00) | 0.4 (0.1, 1.1; 0.08) | 1.0 (0.1, 15.3; 0.98) |

NA: Expected count are not met in each cell

Analysis: adjusted for age, sex, religion, wealth status, educational status, physical activity, diet quality, life time alcohol and khat use

| Calander year | 1965 | 1966 | 1974 | 1975 | 1979 | 1980 | 1981 | 1982 | 1983 | [Sep [1984 | Aug 1985] | 1986 | 1987 | 1987 | 1993 |  |  |
| --- | --- | --- | --- | --- | --- | --- | --- | --- | --- | --- | --- | --- | --- | --- | --- | --- | --- |
| Exposure status |  |  |  |  |  |  |  |  |  | The period of famine | | Washout period  [June 1986 - Aug 1987] | |  |  |  |  |
| In-utero |  | | | | | | | | | DOB  [Oct 1984 - May1986] | | |  |  |  |  |  |
|  |  |  |  |  |  |  |  |  |  |  |  |  |  |  |  |  |  |
| Early childhood,  (0-2 year) |  | | | | | | | DOB  [Oct [1982 - Aug: 1985] | | | |  |  |  |  |  |  |
|  |  |  |  |  |  |  |  |  |  |  |  |  |  |  |  |  |  |
| Preschool  (2-5 year) |  | | | | DOB  [Oct 1979 - Sep 1982] | | |  |  |  |  |  |  |  |  |  |  |
|  |  |  |  |  |  |  |  |  |  |  |  |  |  |  |  |  |  |
| Late childhood  (5-10 year) |  | | DOB  [Oct 1974 - Sep 1979] | | | |  |  |  |  |  |  |  |  |  |  |  |
|  |  |  |  |  |  |  |  |  |  |  |  |  |  |  |  |  |  |
| Adolescence  (10-18 year) |  | DOB  [Oct 1966 - Sep 1974] | | |  |  |  |  |  |  |  |  |  |  |  |  |  |
|  |  |  |  |  |  |  |  |  |  |  |  |  |  |  |  |  |  |
| Conceived and born post-famine |  | | | | | | | | |  |  |  | DOB  [Sep 1987- Dec 1993] | | |  |  |
|  |  |  |  |  |  |  |  |  |  |  |  |  |  |  |  |  |  |

**Supplementary Figure 1. Distribution of date of birth (DOB) and chronological age at famine exposure**

Famine exposed recruited from relocated survivors in the study area

545

Matched controls recruited from native residents in the study area

283

Born post-famine from famine exposed parents recruited in the study area

104

Born post-famine from famine non-exposed parents recruited in the study area

54

Total enrolled

828

Total enrolled

158

Total enrolled

986

Not consented:

20 (3 post-famine +17 exposed)

Included to the survey

966

Analysed

966

***Supplementary Figure 2. Recruitment flow chart of study participants***
