# Supplementary material for: Forty years later: adult health and non-communicable disease following the 1984–1985 Great Ethiopian Famine – a retrospective cohort study
Source: BMJ Glob Health. 2026 Feb 23;11(2):e021721. doi: 10.1136/bmjgh-2025-021721 (PMC12931544; doi:10.1136/bmjgh-2025-021721)
Supplement: online supplemental file 2 [file bmjgh-11-2-s002.docx]

### BMJ Global Health Author Reflexivity Statement

Adapted from Morton, B., Vercueil, A., Masekela, R., Heinz, E., Reimer, L., Saleh, S., Kalinga, C., Seekles, M., Biccard, B., Chakaya, J., Abimbola, S., Obasi, A. and Oriyo, N. (2022), Consensus statement on measures to promote equitable authorship in the publication of research from international partnerships. Anaesthesia, 77: 264-276. <https://doi.org/10.1111/anae.15597>

| **Study conceptualisation** | |
| --- | --- |
| 1. How does this study address local research and policy priorities? | The study was codesigned by involving local stakeholder and the result directly addressing national research gaps. The findings support key policy priorities in NCD prevention, maternal–child nutrition, and targeted interventions for vulnerable populations. |
| 1. How were local researchers involved in study design? | Co-design the idea, lead the local project development, lead the local implementation, analysis and writeup and dissemination. |
| **Research management** | |
| 1. How has funding been used to support the local research team(s)? | Through mentorship, training, scientific authorship, and international exchange experiences |
| **Data acquisition and analysis** | |
| 1. How are research staff who conducted data collection acknowledged? | We acknowledged them by providing them capacity building training, covering cost of their time, respected, understood, and valued. |
| 1. How have members of the research partnership been provided with access to study data? | We have developed data storage, sharing, and use guideline where all member of the research team will have access for the data for ethical and responsible use. |
| 1. How were data used to develop analytical skills within the partnership? | Senior data analysis experts such as Tim J cole, Charles Opondo and Jonathan Wells provided active support and skill transfer during data analysis. |
| **Data interpretation** | |
| 1. How have research partners collaborated in interpreting study data? | All collaborators have been attended biweekly virtual meetings whereby the lead author present and collect feedback on data interpretation. Manuscript has also been circulated and collaborators provided their feedback on interpretation in written form. |
| **Drafting and revising for intellectual content** | |
| 1. How were research partners supported to develop writing skills? | The guided write up by giving feedback and comments on meetings and also directly contributed their inputs on the shared manuscripts and also shared their comments and feedback. |
| 1. How will research products be shared to address local needs? | We share the result for local community, local administrators, national government bodies through presentations and sharing policy brief. |
| **Authorship** | |
| 1. How is the leadership, contribution and ownership of this work by LMIC researchers recognised within the authorship? | The lead author has been leading the project in the local setting (LMICs) and also lead the analysis and write up of the work. |
| 1. How have early career researchers across the partnership been included within the authorship team? | We involved postdoc (the lead authors, PHD student (second author) and other masters students in this project to support early career researchers develop their career trajectory. |
| 1. How has gender balance been addressed within the authorship? | We have considered this and about half of them are males/females. |
| **Training** | |
| 1. How has the project contributed to training of LMIC researchers? | The project supported short term lipidomic and metabolomic training for researchers in LMICS and also supported PHD and MSc students. |
| **Infrastructure** | |
| 1. How has the project contributed to improvements in local infrastructure? | We have secured several research equipment and imported them to Jimma for current and future use. |
| **Governance** | |
| 1. What safeguarding procedures were used to protect local study participants and researchers? | Obtaining informed consent, ensuring confidentiality, and conducting interviews in private and safe settings. Researchers were trained on ethical conduct, referral pathways, and protocols for managing distress or risks, with clear mechanisms to protect both participants and field staff throughout the study. |
